# The novel two-component system AmsSR governs alternative metabolic pathway usage in *Acinetobacter baumannii*

**DOI:** 10.3389/fmicb.2023.1139253

**Published:** 2023-04-04

**Authors:** Leila G. Casella, Nathanial J. Torres, Brooke R. Tomlinson, Mark Shepherd, Lindsey N. Shaw

**Affiliations:** ^1^Department of Cell Biology, Microbiology and Molecular Biology, University of South Florida, Tampa, FL, United States; ^2^School of Biosciences, University of Kent, Canterbury, United Kingdom

**Keywords:** regulation, two component system, *Acinetobacter baumannii*, metabolism, stress response

## Abstract

In this study, we identify a novel two-component system in *Acinetobacter baumannii* (herein named AmsSR for regulator of alternative metabolic systems) only present in select gammaproteobacterial and betaproteobacterial species. Bioinformatic analysis revealed that the histidine kinase, AmsS, contains 14 predicted N-terminal transmembrane domains and harbors a hybrid histidine kinase arrangement in its C-terminus. Transcriptional analysis revealed the proton ionophore CCCP selectively induces P*_amsSR_* expression. Disruption of *amsSR* resulted in decreased intracellular pH and increased depolarization of cytoplasmic membranes. Transcriptome profiling revealed a major reordering of metabolic circuits upon *amsR* disruption, with energy generation pathways typically used by bacteria growing in limited oxygen being favored. Interestingly, we observed enhanced growth rates for mutant strains in the presence of glucose, which led to overproduction of pyruvate. To mitigate the toxic effects of carbon overflow, we noted acetate overproduction in *amsSR*-null strains, resulting from a hyperactive Pta-AckA pathway. Additionally, due to altered expression of key metabolic genes, *amsSR* mutants favor an incomplete TCA cycle, relying heavily on an overactive glyoxylate shunt. This metabolic reordering overproduces NADH, which is not oxidized by the ETC; components of which were significantly downregulated upon *amsSR* disruption. As a result, the mutants almost exclusively rely on substrate phosphorylation for ATP production, and consequently display reduced oxygen consumption in the presence of glucose. Collectively, our data suggests that disruption of *amsSR* affects the function of the aerobic respiratory chain, impacting the energy status of the cell, which in turn upregulates alternative metabolic and energy generation pathways.

## Introduction

*Acinetobacter baumannii* is a Gram-negative pathogen commonly associated with life threating diseases, including urinary-tract infections, ventilator-associated pneumonia, meningitis, and septicemia. Such infections are common in patients from healthcare facilities, and primarily those in intensive care units (Davis et al., 2005; Guerrero et al., 2010; Doyle et al., 2011; Tsakiridou et al., 2014). Resistance among clinical isolates is becoming increasingly common (Mera et al., 2010; Morfin-Otero et al., 2013), leading to recurrent isolation of pan-resistant strains on a global scale (Okpara and Maswoswe, 1994; Zarrilli et al., 2004; Hujer et al., 2006; Carvalho et al., 2009; Clark et al., 2016). As a result, the Worldwide Health Organization has categorized *A. baumannii* in the highest priority group of pathogens, who are defined as being the most critical for new antimicrobial drug development (Willyard, 2017).

During infection, bacterial pathogens encounter myriad unfavorable conditions, including osmotic-, heat-, acid-, and oxidative-stress, nutrient limitation, and host antimicrobial peptides (Fiester and Actis, 2013; Fang et al., 2016). Under these conditions, survival is dependent on an adaptive response controlled by regulatory elements, such as transcription factors, σ-factors, and two-component systems (TCS; Vicente et al., 1999; Boor, 2006; Laub and Goulian, 2007). Of these regulatory elements, TCS are widely employed by bacteria to sense and respond to environmental stimuli (Alm et al., 2006). These systems typically consist of a membrane sensor histidine kinase (HK) that, upon perceiving external insult, autophosphorylates at a conserved histidine residue (Cheung and Hendrickson, 2010; Bhate et al., 2015). This phosphate is then transferred to a conserved aspartate residue on its cytoplasmic response regulator (RR) partner, which is typically a DNA binding protein (Mascher, 2006; Mascher et al., 2006). This signal transduction cascade leads to the coordinated activation or repression of genes required for a wealth of responses, including biofilm formation, motility, and the expression of adherence proteins, exotoxins, efflux pumps and outer membrane porins (Mitrophanov and Groisman, 2008).

Recently, we identified the entire proteinaceous regulome of *A. baumannii*, including 14 TCS conserved within the genomes of numerous clinical isolates (Casella et al., 2017). To date, only six have been characterized: BfmSR, PmrAB, AdeRS, BaeSR, Ompr-EnvZ and GacSA (Dorsey et al., 2002; Marchand et al., 2004; Arroyo et al., 2011; Lin et al., 2014; Liou et al., 2014; Tipton and Rather, 2016). Of these, the first to be discovered was AdeRS, which mediates resistance to fluoroquinolones, chloramphenicol, tigecycline and aminoglycosides (Marchand et al., 2004; Yoon et al., 2013, 2015). It is also responsible for the upregulation of efflux pump expression (Wen et al., 2017), and genes involved in biofilm formation and virulence (Richmond et al., 2016). BaeSR influences efflux pump expression, while also mediating resistance to tannic acids (Henry et al., 2012; Lin et al., 2014, 2015). Additionally, BaeSR is thought to engage in, and control, cross talk between TCS (Lin et al., 2014). PmrAB controls the expression of genes involved in surface charge and outer membrane permeability (Raetz et al., 2007; Arroyo et al., 2011). Consequently, mutations are frequently found in *pmrAB* that impact resistance to last resort antimicrobials, the polymyxins (Adams et al., 2009). BfmSR does not control antibiotic resistance, but instead influences biofilm formation *via* regulation of pilus (Tomaras et al., 2008) and polysaccharide production (Geisinger and Isberg, 2015). GacSA controls numerous processes, including carbon metabolism, motility, pili synthesis and biofilm formation (Dorsey et al., 2002; Cerqueira et al., 2014). Indeed, due to its pleiotropic influence, it is apparent that GacSA serves as a global regulator of virulence (Cerqueira et al., 2014; Bhuiyan et al., 2016). Finally, the least characterized *A. baumannii* TCS Ompr-EnvZ controls colony opacity switching, and virulence in a *Galleria mellonella* model of infection.

Herein, we characterize one of the unstudied TCS, ABUW_2426 (RR) and ABUW_2427 (HK; named AmsSR). Analysis revealed that the proton ionophore carbonyl cyanide *m*-chlorophenyl hydrazone (CCCP) induces P*_amsSR_*. Disruption of *amsSR* leads to decreased membrane polarity and acidification of the cytoplasm. Transcriptomic profiling of an *amsR* mutant revealed altered expression of genes involved in metabolism, leading to an abundance of metabolites produced from an overactive glyoxylate shunt, and increased activity of energy generation pathways typically used in oxygen limited situations. Collectively, our data identifies AmsSR as an important new component of *A. baumannii* regulatory circuits, governing alternative metabolic pathways.

## Materials and methods

### Bioinformatic analyses

Bioinformatic analysis were performed using Pfam and Protter. Acinetobacter species information was retrieved from the List of Prokaryotic names with Standing in Nomenclature (LPSN) website. A search for AmsS homologs was performed at the Ensembl Bacterial and Uniprot using their BLAST function. ArcB sequences were retrieved from the NCBI ftp server. Alignments and protein relatedness assessments were generated using Qiagen CLC Main Workbench (v21.0.1). Percent identity tables were produced from ClustalW alignment data at the EMBL-EBI website.

### Strains and growth conditions

Strains and cloning primers used are listed in Table 1. All strains were cultured in lysogeny broth (LB), unless otherwise indicated, with shaking at 37°C. When appropriate tetracycline (5 μg/mL) and hygromycin (140 μg/mL) were added to media. Mutants of *amsR* and *amsS* were acquired from the AB5075 transposon mutant library (Gallagher et al., 2015). Strains were confirmed using primers OL4155/OL4156 (*amsS*) or OL4157/OL4158 (*amsR*). Unless stated otherwise, synchronous cultures were prepared as follows: *A. baumannii* strains were grown in LB overnight at 37°C with shaking. These cultures were diluted 1:100 into 100 mL fresh LB, grown to exponential phase, before seeding new 100 mL cultures at OD_600_ 0.05.

**Table 1:** Bacteria strains, plasmids, and cloning primers.

Strain	Description	Source
***Escherichia coli***		
DH5α	Cloning strain	Salisbury et al. (1972)
***Acinetobacter baumannii***		
AB5075	Wild-type strain	Jacobs et al. (2014)
AB5075 178::T26	AB5075 with transposon insertion in ABUW_2426 (*amsR*)	This study
AB5075 156::T26	AB5075 with transposon insertion in ABUW_2427 (*amsS*)	This study
LGC2463	AB5075 containing pSLG2 (P*_amsSR_*–*lacZ* fusion)	This study
LGC2591	AB5075 178::T26 complemented with pMQ557::*amsR*	This study
LGC2592	AB5075 156::T26 complemented with pMQ557::*amsS*	This study
**Plasmids**		
pMQ557	Cloning vector for complementation	Gift, Dr. R. Shanks, University of Pittsburgh
pAZ106	Promotorless *lacZ* insertion vector	Kemp et al. (1991)
pSLG1	pAZ106::P*_amsSR_*–*lacZ*	This study
pSLG2	pMQ557::P*_amsSR_*–*lacZ*	This study
pSLG3	pMQ557::*amsR*	This study
pSLG4	pMQ557::*amsS*	This study
**Primers**		
OL4155	ATGCCCGGGGGTAATCAAGTCAAGGTCGA	This study
OL4156	ATGCCCGGGTTAGTGGTGGTGGTGGTGGTGTTGCTTATCCTGAATCAATC	This study
OL4158	ATGCCCGGGTTAGTGGTGGTGGTGGTGGTGGTGAAACTCTTGCACCAAGC	This study
OL4169	ATGGGATCCATAACCGCTGCCCACCATGCT	This study
OL4170	ATGGAATTCTACGACAATTGCTGCCCATA	This study
OL4230	ATGCTCGAGATAACCGCTGCCCACCATGCT	This study
OL4232	ATGGCGGCCGCGACCCAACGCTGCCCGAGAAA	This study

Underscore indicates restriction enzyme site.

### Construction of P*_amsSR_*-*lacZ* transcriptional fusion

A PCR fragment was generated that began ~1 kb 5′ of the *amsS* start codon and ended ~300 bp 3′ of it, using primers OL4169/OL4170. This was cloned into pAZ106, which contains a promoter-less *lacZ* cassette, and transformed into *Escherichia coli* DH5α creating pSLG1. The P*_amsSR_*-*lacZ* fusion was amplified from this using primers OL4230/OL4232, and subsequently cloned into the *A. baumannii* shuttle vector pMQ557, creating pSLG2. AB5075 was then transformed with pSLG2, and colonies were selected using LB plates supplemented with hygromycin (plasmid encoded). All strains were confirmed by PCR.

### Construction of complementation strains

The *amsSR* genes overlap by 4 bp (Supplementary Figure S1), suggesting they are transcriptionally linked, and expressed from a single promoter 5′ of *amsS*. Thus, to complement *amsS*, a 3,979 bp fragment was generated that began 500 bp 5′ of the *amsS* start codon and concluded at the end of the *amsS* open reading frame, using primers OL4155/OL4156. For *amsR* complementation, a 4,945 bp fragment was generated using the same forward primer (OL4155), and a reverse primer (OL4158) that concluded at the end of the *amsR* reading frame. For simplicity, we refer to the *amsR* complement as *amsR*^+^ (rather than *amsSR*^+^). These PCR products were cloned into pMQ557 and transformed into *E. coli* DH5α, creating plasmids pSLG4 and pSLG3, respectively. Clones were confirmed by PCR and Sanger sequencing, before being transformed into the relevant mutants. Strains were again confirmed by PCR and Sanger sequencing.

### β-galactosidase assays

**Figure 1:**
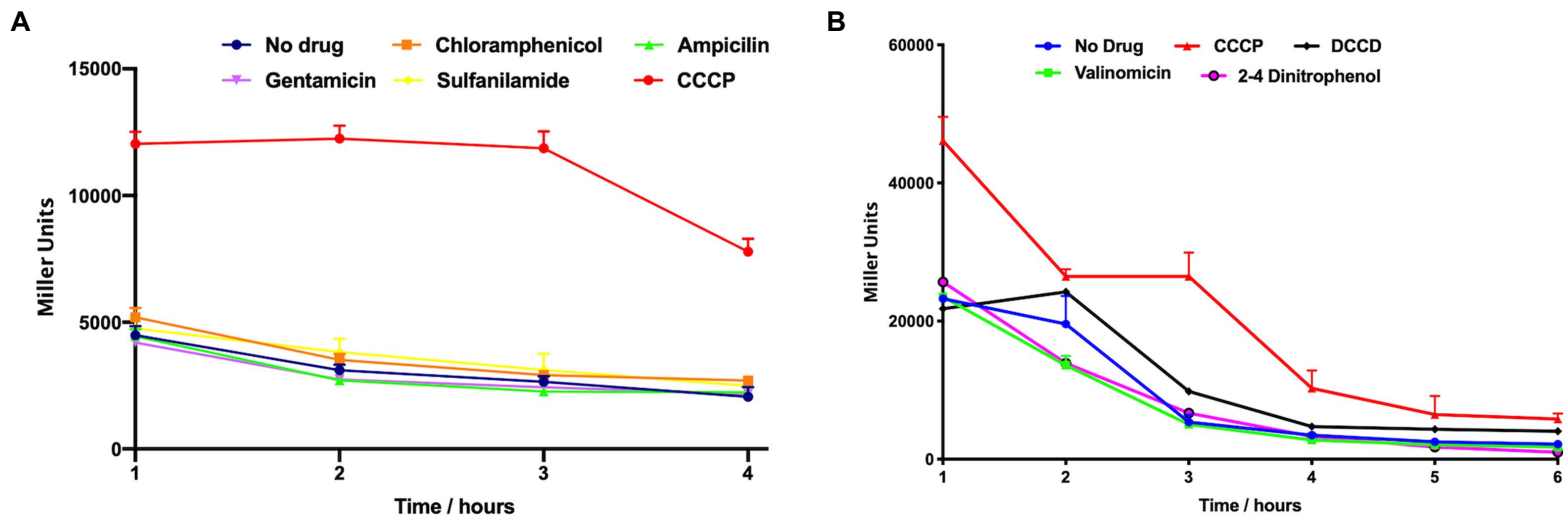
P*_amsSR_* transcription is induced upon exposure to the protonophore CCCP. ß-galactosidase activity of an *amsSR*-*lacZ* fusion strain was measured hourly during exposure to the agents shown. Transcriptional activity is expressed as Miller Units. Data is derived from three independent biological replicates, with panels **(A)** and **(B)** generated from separate experiments. Error bars ± SEM.

A plate-based screening assay was performed as previously described (Shaw et al., 2008). Briefly, LB plates were overlaid with 5 mL of LB top agar (0.7%) supplemented with X-Gal (40 μg/mL) and the *A. baumannii* P*_amsSR_*-*lacZ* strain. Sterile disks were placed on plates and each disk was inoculated with 10 μL of a chemical stressor. Plates were incubated overnight at 37°C and induction of expression was recorded as a blue halo around filter discs. For expression over time, synchronized cultures were prepared in LB as detailed above, before the following compounds were added: 12.5 μg/mL Chloramphenicol, 100 μg/mL Ampicillin, 100 μg/mL Gentamicin, 100 μg/mL Sulfanilamide, 6.25 μg/mL CCCP, 100 μg/mL N’N′ dicyclohexylcarbodiimide (DCCD), 25 μg/mL Valinomycin, and 75 μg/mL 2–4 dinitrophenol (DNP). Cultures were incubated with shaking at 37°C, with 0.1 mL samples collected hourly. β-galactosidase activity was measured as described previously (Miller et al., 2012). Results are the average of three biological replicates; with data from Figures 1A,B generated from separate experiments.

### Intracellular pH measurement

Synchronous cultures were prepared in Mueller Hinton Broth (MHB) before being centrifuged at 4,500 g for 10 min and washed with 50 mM potassium phosphate buffer, pH 7.0, supplemented with 5 mM EDTA. Cells were pelleted and resuspended in the same buffer, followed by the addition of the BCECF-AM dye [2′,7’-Bis-(2-Carboxyethyl)-5-(and-6)-Carboxyfluorescein, Acetoxymethyl Ester] at a final concentration of 20 μM. Samples were incubated for 30 min at room temperature. After this time, cells were pelleted, washed, and resuspended in the same buffer, followed by addition of glucose to a final concentration of 10 mM. Samples were incubated for 5 min at 37°C, before 200 μL aliquots were withdrawn and added to the wells of a 96-well plate. Fluorescent signals were recorded for 20 min at an excitation of 490 nm and emission of 530 nm using a BioTek Synergy II plate reader. As a control, 6.25 μg/mL CCCP was added to cultures after 10 min to decrease intracellular pH. To create a calibration curve, cells were resuspended in high potassium buffers (135 mM KH_2_PO_4_/20 mM NaOH and 110 mM K_2_HPO_4_/20 mM NaOH) with a pH range from 6.5 to 8.0. Results are the average of three biological replicates.

### Membrane depolarization assay

Membrane depolarization was measured using the membrane potential sensitive fluorescent dye 3,3′-Dipropylthiadicarbocyanine Iodide [DiSC3 (5)]. Synchronous cultures were prepared as above before being harvested by centrifugation and washed thrice with 5 mM HEPES buffer, pH 7.2, containing 5 mM glucose. Samples were resuspended to OD_600_ 0.05 in the same buffer. Cell suspensions were incubated with 100 mM KCl and 2 μM of DISC3 for 15 min at room temperature, before being added to the wells of 96-well plates. Fluorescence was monitored at an excitation wavelength of 662 nm and an emission wavelength of 670 nm. Reads were taken 30 min after reactions started. Results are the average of three biological replicates.

### RNA sequencing and bioinformatic analysis

RNAseq experiments were performed as described previously (Tomlinson et al., 2022). Briefly, synchronous cultures of the *A. baumannii* wild-type and *amsR* mutant were prepared in biological triplicate. After growth for 1 h, CCCP was added at a concentration of 6.25 μg/mL. Cultures were grown for 2 h, before 5 mL was harvested from each flask. This was added to 5 mL of ice-cold PBS, pelleted by centrifugation at 4°C, and the supernatant removed. Total RNA was isolated using a RNeasy Kit (Qiagen) and DNA was removed using a TURBO DNA-free kit (Ambion). Sample quality was assessed using an Agilent 2100 Bioanalyzer with an RNA 6000 Nano kit (Agilent). An RNA integrity (RIN) value of >9.7 was used as a cutoff. Prior to mRNA enrichment, triplicate biological RNA samples were pooled at equal concentrations followed by rRNA removal using a Ribo-Zero Kit for Gram-negative bacteria (Illumina) and a MICROBExpress Bacterial mRNA enrichment kit (Agilent). Removal efficiency of rRNA was confirmed as for RIN. Library preparation was performed using the TruSeq Stranded mRNA Kit (Illumina) omitting mRNA enrichment. Quality, concentration, and average fragment size was assessed with an Agilent TapeStation and High Sensitivity DNA ScreenTape kit. Library concentration for the pooling of barcoded samples was assessed by qPCR using a KAPA Library Quantification kit (KAPA Biosystems). Samples were run on an Illumina MiSeq with a 150-cycle MiSeq Reagent kit. Data was exported from BaseSpace (Illumina) in fastq format and uploaded to Qiagen Bioinformatics for analysis. Data was aligned to the AB5075 reference genome (NZ_CP008706.1), and experimental comparisons were carried out after quantile normalization using the experimental fold change feature.

### Quantitative real time PCR

Quantitative real time PCR analyses were conducted as described previously, using primers in Supplementary Table S1 (Tomlinson et al., 2022). Bacterial cultures were grown, and RNA extracted as described for RNA-seq experiments. Results are the average of three biological replicates.

### Growth in glucose assays

Overnight cultures were synchronized and grown for 3 h in M9 media supplemented with 1% casamino acids. These cultures were used to standardized cultures to OD_600_ 0.05 in M9 media supplemented with glucose to a final concentration of 0.2%. OD_600_ was read every 2 h; results are the average of three biological replicates.

### Intracellular metabolite quantification assays

To measure intracellular metabolite concentrations strains were synchronized and grown for 3 h in LB. After 3 h, cells were harvested and lysed using a Mini BeadBeater-16 (Biospec). For acetate, pyruvate and succinate quantification, cells were resuspended in 100 μL of their assay buffer (Biovision) before lysis. For pyruvate quantification, cells were not lysed, but instead were pelleted and pyruvate extracted with 4 volumes of pyruvate assay buffer (Biovision). For NADH^+^ assessment, cells were not lysed, but instead were pelleted and total NADH (NADH and NAD) extracted with 400 μL extraction buffer (Biovision). NAD was decomposed by incubating samples for 30 min at 60°C. Intracellular metabolite quantification was then performed using: an acetate-, pyruvate-, oxaloacetate-, succinate-, or NADH-colorimetric assay kit (all Biovision), as per the manufacturer’s protocol. Results are the average of three biological replicates.

### Extracellular glucose measurement

To measure extracellular glucose strains were synchronized and grown for 3 h in LB media supplemented with glucose to a final concentration of 0.2%. After 3 h, 50 μL of culture supernatant was withdrawn and added to wells of a 96-well plate. Glucose concentrations in samples was quantified using a glucose colorimetric assay kit (Biovision) following the manufacture’s protocol. Results are the average of three biological replicates.

### Cellular ATP assays

Strains were synchronized and grown for 3 h in LB media. Following 1 h of growth, CCCP was added at a concentration of 6.25 μg/mL and cultures were grown for 2 h. Cells were washed and pelleted, resuspended in HEPES buffer, and incubated on ice for 30 min. Pyruvate (10 mM) was added to samples, followed by incubation at 30°C for 30 min. Cells were collected and resuspended in ATP buffer (Biovision), lysed using a Mini BeadBeater-16, and ATP quantification performed using an ATP colorimetric assay kit (Biovision) following the manufacturer’s protocol. Results are the average of three biological replicates.

### Extracellular oxygen consumption

Oxygen consumption was measured using an extracellular oxygen consumption kit (Abcam) following the manufacturer’s instructions. Briefly, overnight cultures were synchronized and grown for 3 h in M9 media supplemented with 1% casamino acids. These were then used to standardized cultures to OD_600_ 0.1 in the same media with or without 0.2% glucose. Standardized cultures (150 μL) were mixed with 10 μL of oxygen consumption reagent in a black 96-well plate and sealed with 100 μL mineral oil. Extracellular oxygen consumption was monitored using a Cytation 5 plate reader (BioTek) for 2 h in 1 min 40s intervals (excitation = 380 nm; emission = 645 nm) at 37°C. The rate of oxygen consumption was calculated by determining slope values for each sample after 30 min. Results are the average of three biological replicates.

### Data availability

Raw RNA sequencing data can be found at the NCBI Gene Expression Omnibus database under GEO Accession GSE123635.

## Results

### Identification of the novel TCS AmsSR

**Figure 2:**
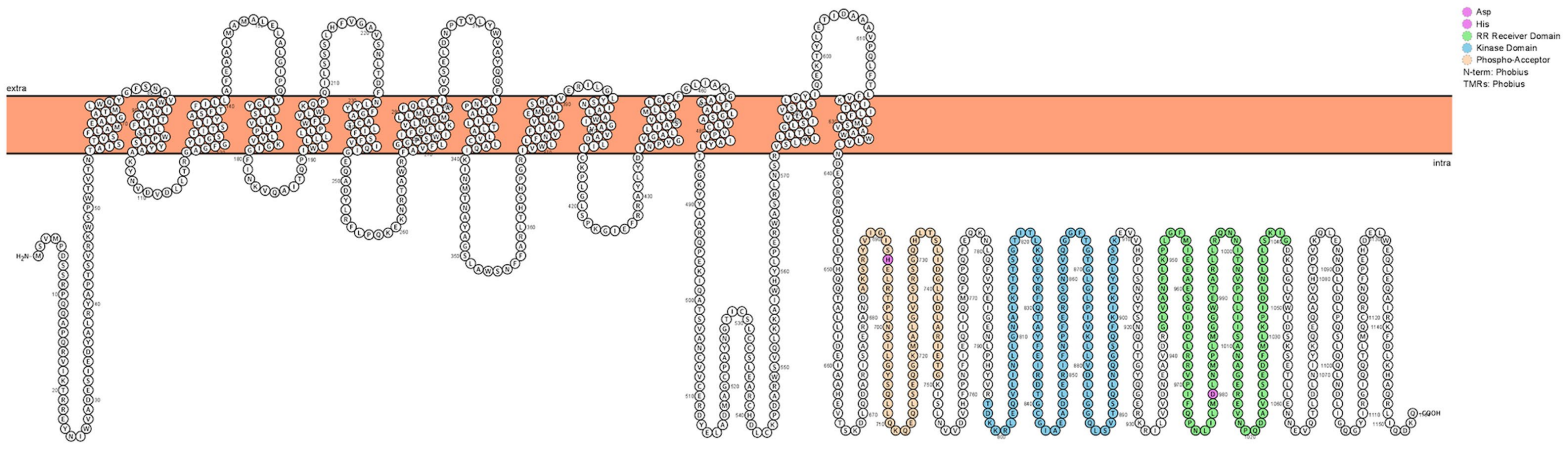
AmsS has a Unique Predicted Structure for a Histidine Kinase. Shown is a predicted topology plot for AmsS generated using Protter. The kinase domain is shown in blue, the phospho-acceptor domain in orange, and the response regulator receiver domain in green. Conserved histidine and aspartate residues are shaded in pink.

Previously, our group performed a global analysis of the *A. baumannii* genome, identifying its complete set of regulatory factors (Casella et al., 2017). To extend this work, we focused our attention on the 14 TCS in strain AB5075. While six have been studied, the remaining eight have yet to be characterized. Previously, we were able to infer function for 7 of these HK elements bioinformatically. As such, herein we characterized the remaining, cryptic TCS module of *A. baumannii* (ABUW_2426-ABUW_2427). An initial examination of the HK (ABUW_2427) revealed it has a unique predicted structure (Figure 2). Specifically, this protein contains 14 predicted transmembrane domains (TMD) in its N-terminus, which far exceeds the two typically seen in HKs. In the C-terminus, there exists a typical HK ATPase domain (shaded blue) for phosphate generation, and a phospho-acceptor domain (shaded orange) with conserved histidine residue to receive this phosphate. Additionally, there also exists a RR receiving domain (shaded green) with conserved aspartic acid residue. As the name suggests, such a domain is observed in the RR of TCS but can also be present in HK molecules. Such proteins are referred to as hybrid HKs (Ma and Phillips-Jones, 2021). It is common in hybrid HKs that a further domain, a histidine phosphotransfer (Hpt) domain, also exists to receive phosphates from the HK RR receiving domain and transfer these to partner RR proteins. Interestingly, no such domain exists in ABUW_2427, suggesting that an additional Hpt-domain containing protein exists in *A. baumannii* to receive these phosphates. Immediately downstream of ABUW_2427 is ABUW_2426, which encodes a RR of the CitB/NarL family and contains a LuxR-DNA-binding helix-turn-helix domain (Casella et al., 2017). Based on the data generated below, we named this TCS: Regulator of Alternative Metabolic Systems (AmsSR).

### Expression of AmsSR is selectively induced by CCCP

To explore function for AmsSR we set out to examine how its transcription is induced by the cell in response to external stress. As such, a *lacZ*-reporter fusion was created for P*_amsSR_* in the wild-type and used in a plate-based disk diffusion assay detailed by us previously (Kolar et al., 2011). Of the 23 compounds used, which elicited myriad stresses (including, DNA-, oxidative-, osmotic-, detergent-, alkali-, acid-, nitric oxide-, alcohol-, and membrane-stress), we observed only the protonophore CCCP resulted in enhanced P*_amsSR_* expression (Supplementary Figure S2).

To validate this, β-Galactosidase activity was measured over time in the presence or absence of several compounds used in the plate-based screen. Again, we observed that transcription from P*_amsSR_* was low in all conditions other than those containing CCCP (Figure 1A). This is of interest because CCCP has the effect of dissipating the electrochemical gradient by shuttling protons across the cell membrane into the cytoplasm. This results in de-energization of the membrane alongside a decline in proton-coupled ATP production, which changes the energy state of the cell resulting from decreased movement of electrons along the respiratory chain (Heytler, 1963; Cunarro and Weiner, 1975). These changes mirror those that take place when oxygen is absent and bacteria are forced to use alternate metabolic pathways to generate energy (Goldsby and Heytler, 1963; Otten et al., 1999). To explore this more broadly, we next measured P*_amsSR_* activity in the presence of other uncouplers with different mechanism of actions. These included DCCD, an inhibitor of the ATPase subunit F_o_/F_1_ that impedes translocation of H^+^ resulting in arrest of ATP production; valinomycin, a K-ionophore that increases the efflux of K^+^; and DNP, a protonophore that transports H^+^ along the electrochemical gradient. Interestingly, we again observed P*_amsSR_* activity was only stimulated in the presence of CCCP (Figure 1B).

### Disruption of AmsSR reduces intracellular pH and depolarizes membranes

**Figure 3:**
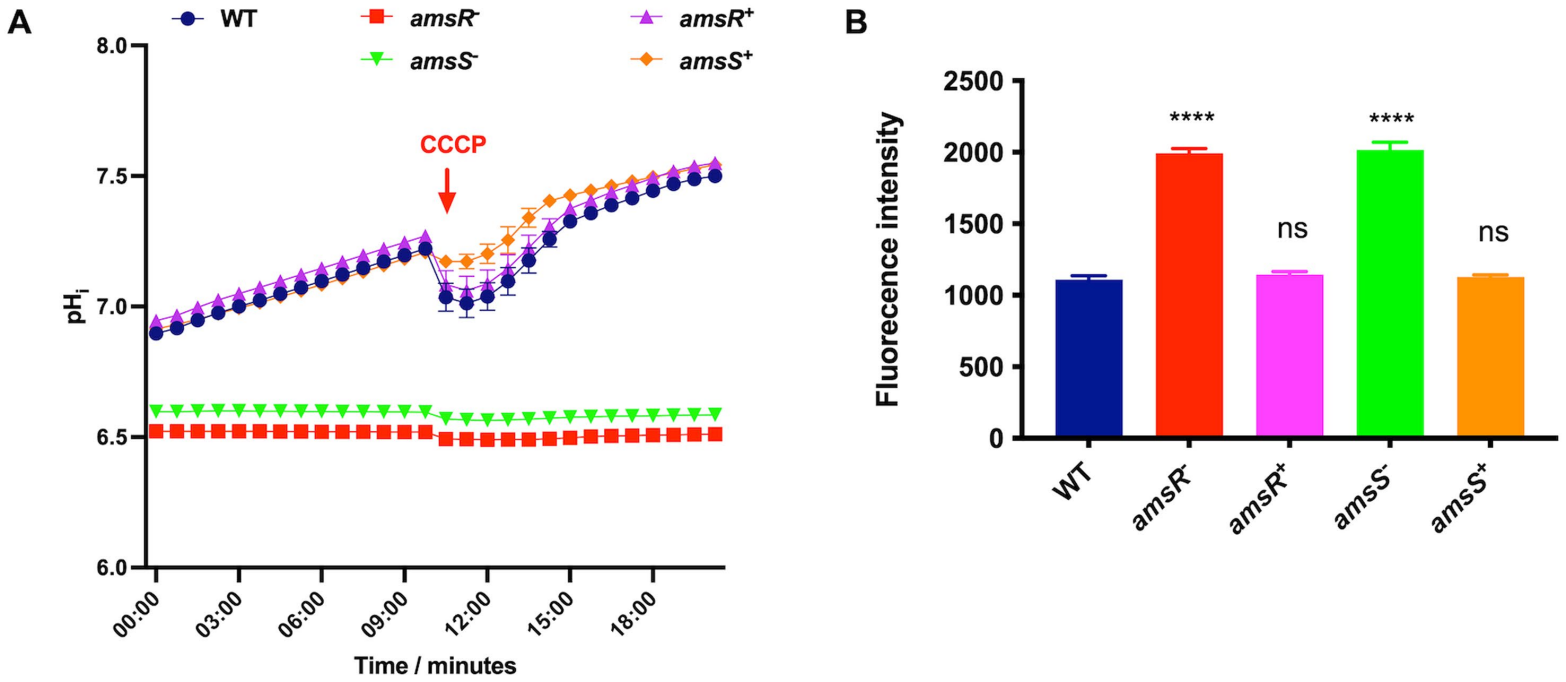
*Acinetobacter baumannii amsSR* mutants have a reduced intracellular pH and depolarized membranes. **(A)** Intracellular pH was measured using the dye BCECF_AM. Exponentially growing cells were treated with the dye alongside the addition of glucose to a final concentration of 10 mM. A red arrow indicates the time at which CCCP was added to all strains. **(B)** Depolarization of cytoplasmic membranes measured by release of the DiSC3 (5) dye. One-way analysis of variance, with Dunnett’s multiple-comparisons test, was used to assess statistical significance in comparison to wild-type, ^****^p < 0.0001. ns, not significant. All data is derived from three independent biological replicates. Error bars ± SEM.

Given that *A. baumannii* induces expression of P*_amsSR_* in the presence of CCCP, we next wanted to determine if deletion of *amsR* or *amsS* resulted in alterations of the transmembrane proton gradient and transmembrane potential, both of which are important for maintenance of an electrochemical gradient across the cell membrane (Taylor, 1983; Mitchell, 2011). Accordingly, we first used the pH sensitive dye BCECF-AM to measure the transmembrane proton gradient. This dye crosses the membrane and is hydrolyzed by esterases to give a fluorescence signal used as a measure of intracellular pH. Upon treatment of the wild-type, *amsR* and *amsS* mutants and complemented strains, alongside an addition of glucose to energize cells, a steady increase in the intracellular pH was observed for the parent and complemented strains. This indicates active pumping of protons across the membrane due to active respiration (Figure 3A). In contrast, the mutants showed no changes in intracellular pH during this analysis, indicating a lack of proton exchange across their membranes. In addition, we also observed a lower intracellular pH for both mutant strains, indicating a higher proton concentration within the cell. This would suggest that mutants lack the ability to pump protons out of the cell, suggesting an ablated transmembrane proton gradient in these strains.

As an additional step, we also added CCCP midway through incubation as a control. This would have the effect of bringing protons across the membrane to decrease the intracellular pH. As expected, a drop in intracellular pH was observed for the wild-type and complemented strains in response to CCCP, indicating these strains can respond to intracellular pH shifts. In contrast, we observed no change in intracellular pH for either mutant upon addition of CCCP. This lack of response is logical given that this molecule requires an energized membrane to produce its H^+^ transport effect; while our mutants appear to have defects in their membrane energetics.

We next sought to measure transmembrane potential using the voltage sensitive dye DiSC3 (5), which accumulates and quenches in negatively charged cytoplasmic membranes. Thus, when membranes are depolarized (less negatively charged) the dye is released, resulting in increased fluorescence. When tested, DiSC3 (5) fluorescence increased in the *amsR and amsS* mutants, compared to the parental and complemented strains (Figure 3B), indicating our mutant strains have a destabilized cellular membrane. This, and the fact that the mutants demonstrate a decrease in internal pH due to a lack of protons being pumped out of the cell, suggest that disruption of *amsSR* hinders energy generation, potentially *via* the TCA cycle or the electron transport chain (ETC).

### Transcriptomic profiling reveals a shift in expression of genes required for glucose utilization upon *amsR* disruption

**Figure 4:**
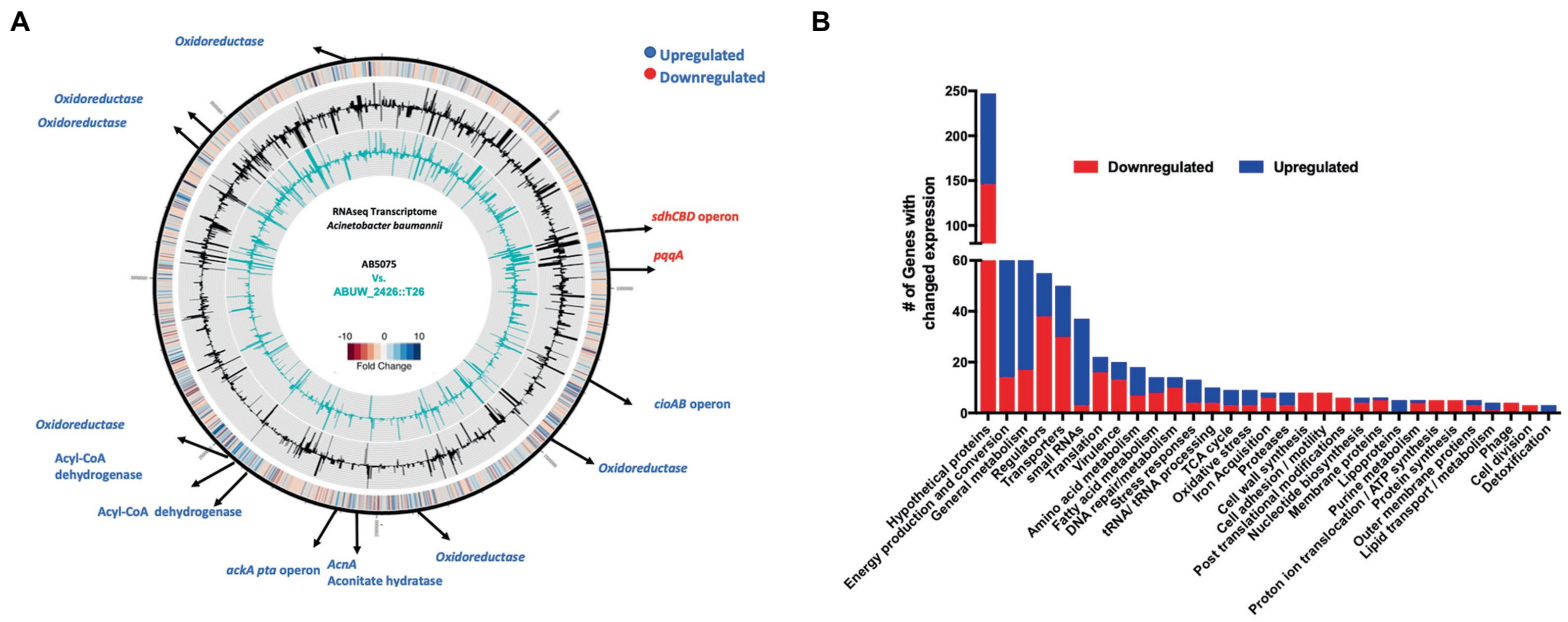
Transcriptomic profiling of the *amsR* mutant. **(A)** Genomic map showing major transcriptional changes in the *amsR* mutant compared to the wild-type after 2 h exposure to CCCP. The outermost circle represents a heat map of changes in *amsR^−^* compared to AB5075. The middle circle (black) depicts RPKM values for AB5075. The inner circle (turquoise) depicts RPKM values for the *amsR* mutant. Black arrows at specific locations highlight genes that function in energy conversion and production. Genes in blue are upregulated and in red are downregulated in *amsR^−^*. **(B)** Categorization of genes differentially expressed in *amsR^−^* compared to the wild-type based on ontology.

To determine the mechanistic role of AmsSR in *A. baumannii*, we next explored its regulon using RNA-seq. Given that transcription from P*_amsSR_* is minimal under standard conditions [above and (Tomlinson et al., 2022)], and that CCCP induces the expression of this system, we performed these experiments using the wild-type and *amsR* mutant grown in the presence of this compound. Upon analysis, we identified 738 genes significantly differentially expressed between the two strains at ≥2-fold (Figure 4A; Supplementary Table S2). This included 357 that were upregulated in the mutant strain, and 381 that were downregulated. These findings were confirmed *via* qRT-PCR for a collection of unrelated genes, demonstrating similar fold changes in all cases (Supplementary Figure S3). When these alterations were parsed ontologically (Figure 4B) we found that most changes were for genes associated with regulatory-, transport-, energy-, or metabolic-functions.

**Figure 5:**
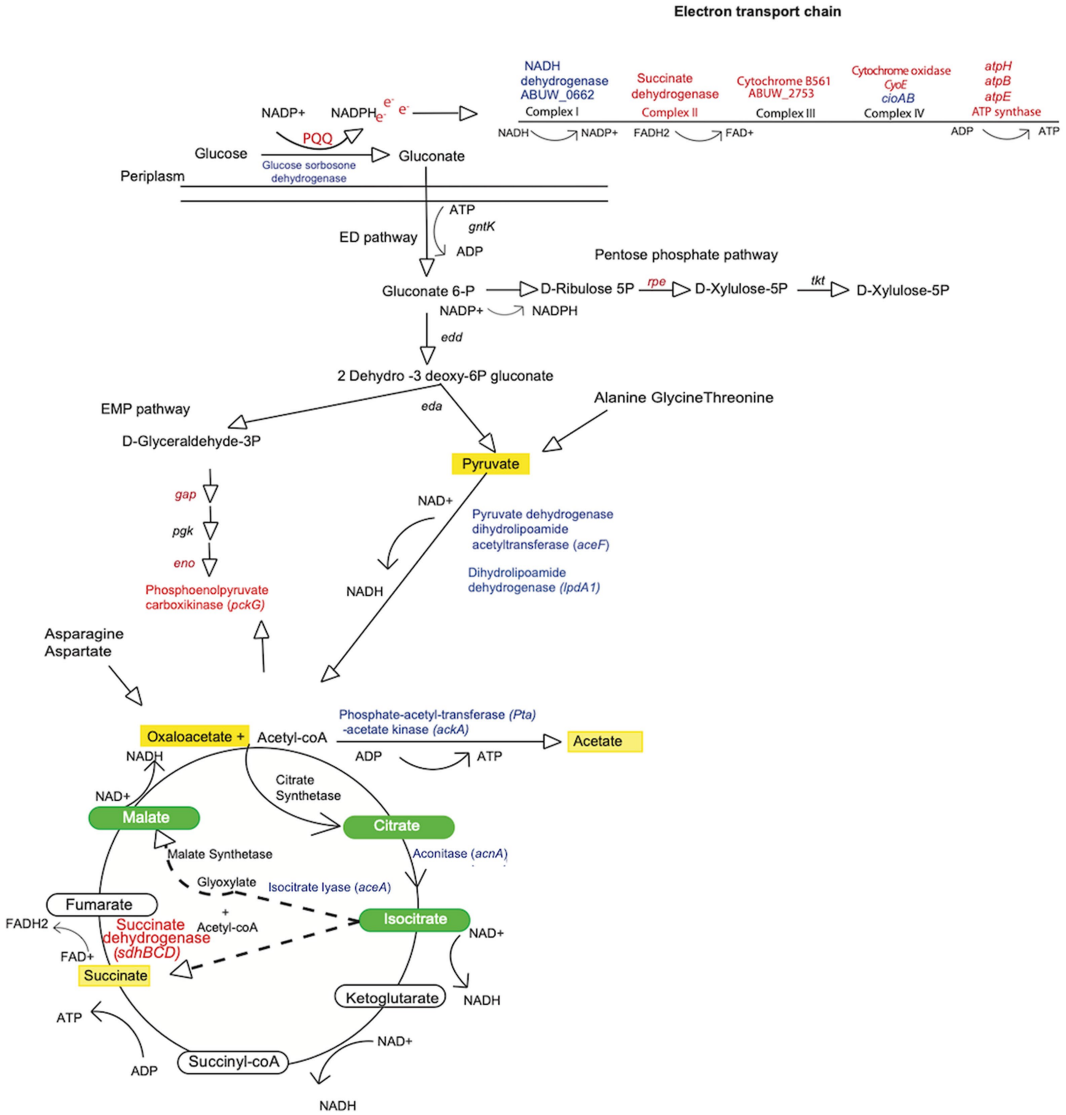
Metabolic map for *Acinetobacter baumannii* indicating nodes of change in the *amsR* mutant. Shown is a metabolic map for *A. baumannii* depicting genes with altered expression from Figure 4A. Blue font indicates genes upregulated in the mutant, while red indicates genes that are downregulated. Green ovals indicate metabolites of the glyoxylate pathway. Dashed arrows indicate the two products (glyoxylate and succinate) formed from isocitrate conversion through the glyoxylate shunt. Yellow shading indicates metabolites that accumulate in the *amsR^−^* strain.

**Figure 6:**
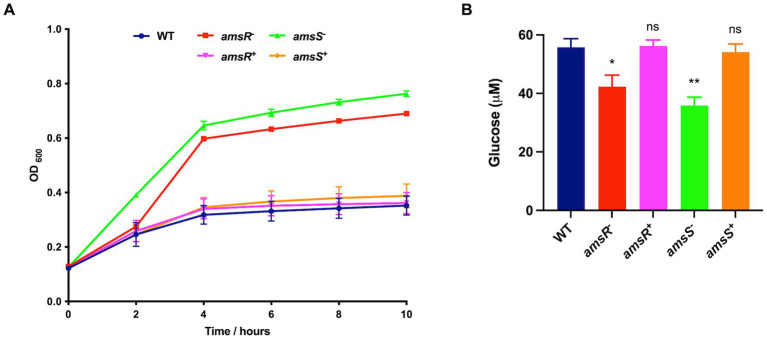
Enhanced glucose uptake in the *amsR* and *amsS* mutant strains results in accelerated growth and intracellular glucose accumulation. **(A)** Growth of strains in M9 media supplemented with 11 mM glucose. **(B)** Extracellular glucose concentrations were measured from exponentially growing cells in LB supplemented with 11 mM glucose. Data is derived from three independent biological replicates. Error bars ± SEM. One-way analysis of variance, with Dunnett’s multiple-comparisons test, was used to assess statistical significance in comparison to wild-type, ^*^p < 0.05; ^**^p < 0.01; ns, not significant.

One of the most striking findings was a 31.8-fold decrease in expression of the Coenzyme PQQ synthesis protein A (*pqqA*) in the *amsR* mutant compared to wild-type. This enzyme is involved in Pyrroquinolone quinone (PQQ) synthesis, which is a required cofactor for the conversion of glucose to gluconate in *Acinetobacter calcoaceticus* (Beardmore-Gray and Anthony, 1986; Goosen et al., 1989). This is important because *A. baumannii* cannot undertake the initial steps of glycolysis as it lacks the enzymes that phosphorylate glucose in the Embden-Meyerhof-Parnas (EMP) pathway (Soares et al., 2009). Instead, it metabolizes gluconate to gluconate-6-P, *via* the Entner Doudoroff (ED) pathway, before processing this further *via* the remaining ED pathway, or the Pentose Phosphate (PP) pathway (Figure 5). From here, this can be fed into the later stages of glycolysis *via* those EMP pathway enzymes that are present in *A. baumannii*. Given the magnitude of this change in *pqqA* expression, one might expect that our mutants would be unable to use glucose as a sole carbon source. To test this, the wild-type, mutant and complemented strains were grown in M9 media supplemented with glucose as a sole carbon source. Strikingly, the mutants exhibited a pronounced growth enhancement compared to the wild-type and complemented strains (Figure 6A). To explore this further, we next determined the extracellular concentration of glucose for our strains (Figure 6B). Here we noted that our mutants had lower extracellular glucose concentrations compared to the wild-type and complemented strains. This indicates that our mutants seemingly have enhanced glucose uptake facilitating faster growth rates.

To understand this, we reviewed our RNAseq dataset for genes with altered expression in the mutant. Here we noted increased expression of a glucose dehydrogenase (ABUW_0055) and a Solute carrier family sodium/glucose transporter (ABUW_0182) in the mutant. Conversely, the remaining genes within the Coenzyme PQQ biosynthetic pathway (*pqqBCDE*) did not display any change in expression upon *amsR* disruption. Thus, it is possible that ABUW_0182 may facilitate enhanced glucose uptake in the mutant, and that the products of *pqqBCDE* are able to generate sufficient levels of PQQ to drive conversion of glucose to gluconate *via* the ABUW_0055 glucose dehydrogenase.

**Figure 7:**
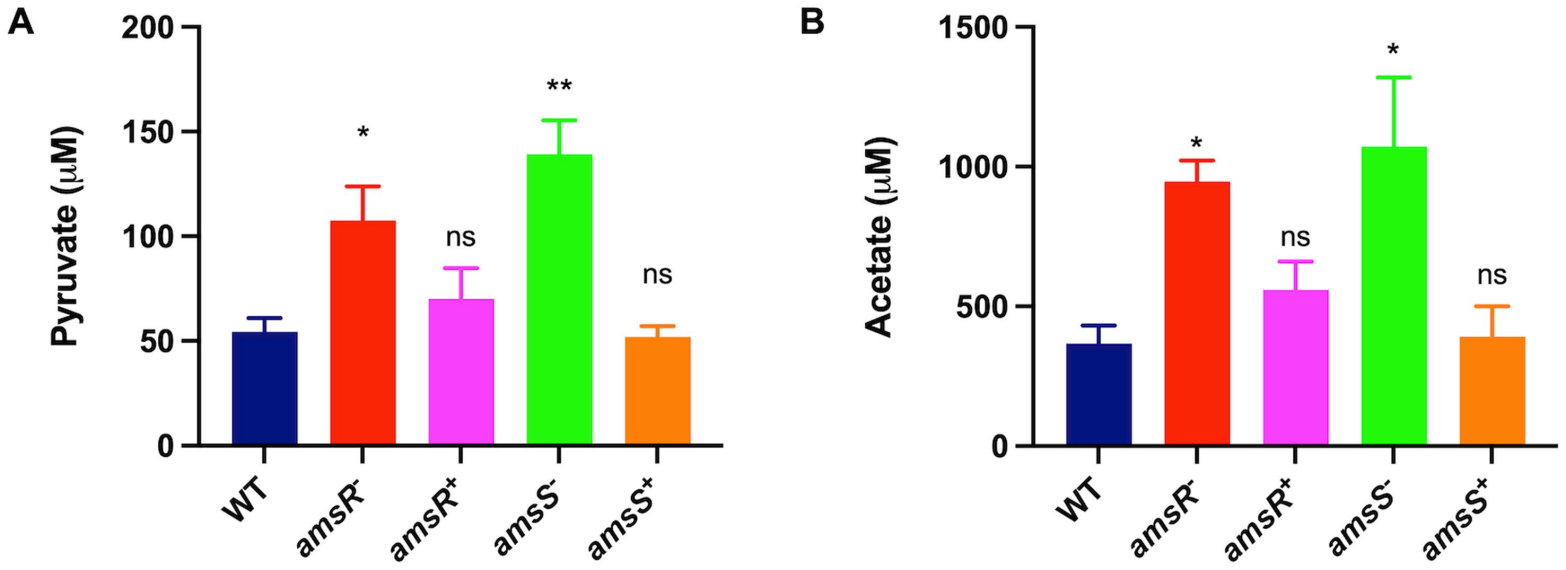
Increased pyruvate in the *amsR* and *amsS* mutant strains lead to a glyoxylate shunt that favors acetate production. Intracellular levels of pyruvate **(A)** or acetate **(B)** were measured in exponentially growing cells. Data is derived from three independent biological replicates. Error bars ± SEM. One-way analysis of variance, with Dunnett’s multiple-comparisons test, was used to assess statistical significance in comparison to wild-type, *p < 0.05, **p < 0.01, ns, not significant.

Interestingly, enzymes in the EMP and PP pathways that would process gluconate-6-P are also downregulated in our mutant. These include glyceraldehyde-3-phosphate dehydrogenase and phosphopyruvate hydratase enolase from the EMP pathway, and ribulose-phosphate 3-epimerase from the PP pathway. This would suggest that our mutant may bypass these metabolic avenues, instead choosing to create pyruvate *via* the ED pathway, which could be fed into the TCA cycle (Figure 5). To test this, we measured the intracellular concentration of pyruvate for our wildtype, mutant and complemented strains (Figure 7A). Here, we noted that our mutants had much higher levels of pyruvate (*amsR*^−^ = 107.46 μM, *amsS*^−^ = 139.19 μM) compared to the wildtype (54.4 μM) and complemented strains (*amsR^+^* = 70.08 μM, *amsS^+^* = 52.01 μM).

### Increased glycolytic influx in the mutant favors activation of the Pta-AckA pathway

Of note, our RNA-seq reveals increased expression of pyruvate dehydrogenase dihydrolipoamide acetyltransferase (*aceF*) and dihydrolipoamide dehydrogenase (*lpdA1*), which form the pyruvate dehydrogenase complex that converts pyruvate to acetyl CoA (Figure 5). This would make sense given that our mutant accumulates pyruvate, and that the generated acetyl-coA could be fed into the TCA cycle to generate energy. Additionally, when bacterial cells grow rapidly in the presence of excessive glucose, the phosphotransacetylase-acetate kinase (Pta-AckA) pathway is induced, favoring conversion of acetyl-CoA to acetate (Wolfe, 2005; Enjalbert et al., 2017) to prevent carbon overflow, which can have toxic effects. Interestingly, we found the *ackA-pta* operon was upregulated in the mutant, thus it is logical that our mutant might prevent carbon overflow, caused by excessive glucose accumulation and consumption, by generating higher levels of acetate. To explore this, the wild-type, mutant and complemented strains were cultured in LB, and cells were collected to measure intracellular acetate levels. As shown in Figure 7B, we observed that the mutants had significantly higher concentrations of acetate (*amsR*^−^ = 946.67 μM, *amsS* ^−^ = 1072.05 μM) than the wild-type (367.41 μM) and complemented strains (*amsR*^+^ = 559.03 μM, *amsS*^+^ = 391.26 μM). Accordingly, it appears that upon disruption of *amsSR* cells produce significantly more pyruvate, which is redirected toward acetate production to prevent the negative effects of excessive carbon flow through the TCA cycle.

### *amsR* mutants favor the glyoxylate shunt

**Figure 8:**
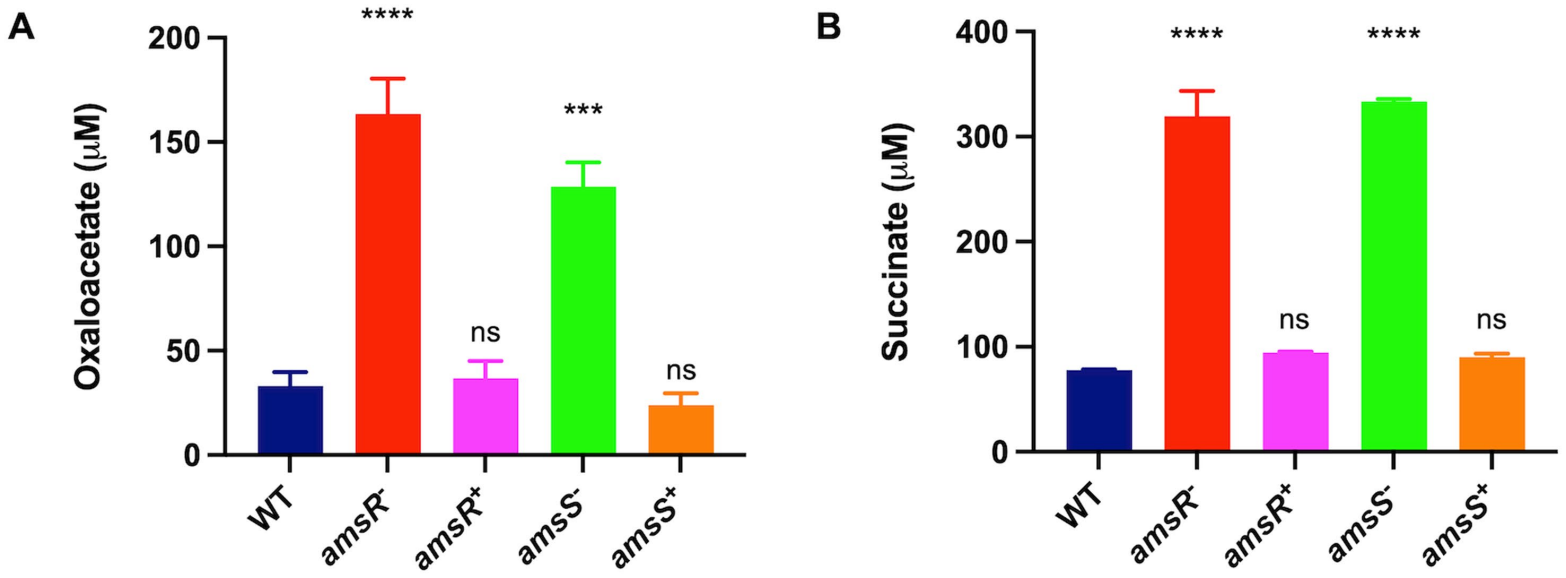
Oxaloacetate and succinate accumulate in *amsSR* mutants due to impaired TCA cycle activity. The concentration of oxaloacetate **(A)** or succinate **(B)** was measured in exponentially growing cells. Data is derived from three independent biological replicates. Error bars ± SEM. One-way analysis of variance, with Dunnett’s multiple-comparisons test, was used to assess statistical significance in comparison to wild-type, ^***^p < 0.001, ^****^p < 0.0001, ns, not significant.

When examining expression of TCA cycle genes, we noted that aconitate hydratase-1 (*acnA*) and isocitrate lyase (*aceA*) were both upregulated in the mutant. This latter gene is of interest as it is used to circumvent the complete TCA cycle, instead facilitating the glyoxylate shunt (Figure 5). This would make sense as we also observed diminished expression of succinate dehydrogenase, which allows for conversion of succinate to fumarate in the TCA cycle. As such, one would predict that our mutant is unable to complete the full TCA cycle and is thus forced into using the glyoxylate shunt to generate energy. Consequently, the malate produced would be converted to oxaloacetate, which in turn would have two fates: Firstly, oxaloacetate can be converted into phosphoenolpyruvate that can be funneled into gluconeogenesis. Importantly, in our mutant, the enzyme phosphoenolpyruvate carboxykinase, which catalyzes this conversion, is downregulated. Secondly, citrate synthetase catalyzes the 1:1 condensation of oxaloacetate and acetyl-CoA to generate citrate, thus continuing the glyoxylate shunt. Because the glyoxylate cycle produces increased oxaloacetate, which can be used for gluconeogenesis, and the enzyme required for this step is downregulated in our mutant, it seems likely that oxaloacetate would accumulate in the mutant strains. To test this, we grew our wild-type, mutants and complemented strains in LB media, and measured oxaloacetate concentrations within cells. Upon analysis, we observed that both mutants had higher amounts of oxaloacetate (*amsR*^−^ = 163.38 μM, *amsS*^−^ = 128.66 μM) than the wildtype (33.16 μM) and complemented strains (*amsR^+^* = 36.7 μM, *amsS^+^* = 23.74 μM; Figure 8A). This confirms that our mutants accumulate oxaloacetate *via* impaired conversion of oxaloacetate to phosphoenolpyruvate.

Of note, the glyoxylate pathway has the side effect of generating succinate as a byproduct. As suggested earlier, the *amsR* mutant has diminished expression of the enzyme that converts succinate into fumarate. Accordingly, we hypothesized that the limited activity of this enzyme would lead to accumulation of succinate within our mutants because of a stalled TCA cycle. Accordingly, the wild-type, mutant and complemented strains were grown in LB media, before extracts were generated and the level of succinate determined (Figure 8B). In so doing, we noted that the *amsR* and *amsS* mutants had much higher levels of succinate (*amsR*^−^ = 319.57 μM, *amsS*^−^ = 333.65 μM). Conversely, succinate levels were lower in the parental (77.73 μM) and complemented strains (*amsR^+^* = 94.35 μM, *amsS^+^* = 90.15 μM). As such, this confirms our hypothesis that a stalled TCA cycle created by decreased succinate dehydrogenase expression elicits succinate accumulation in mutant cells.

### A hyperactive glyoxylate shunt contributes to increased NADH levels in *amsSR* mutants

**Figure 9:**
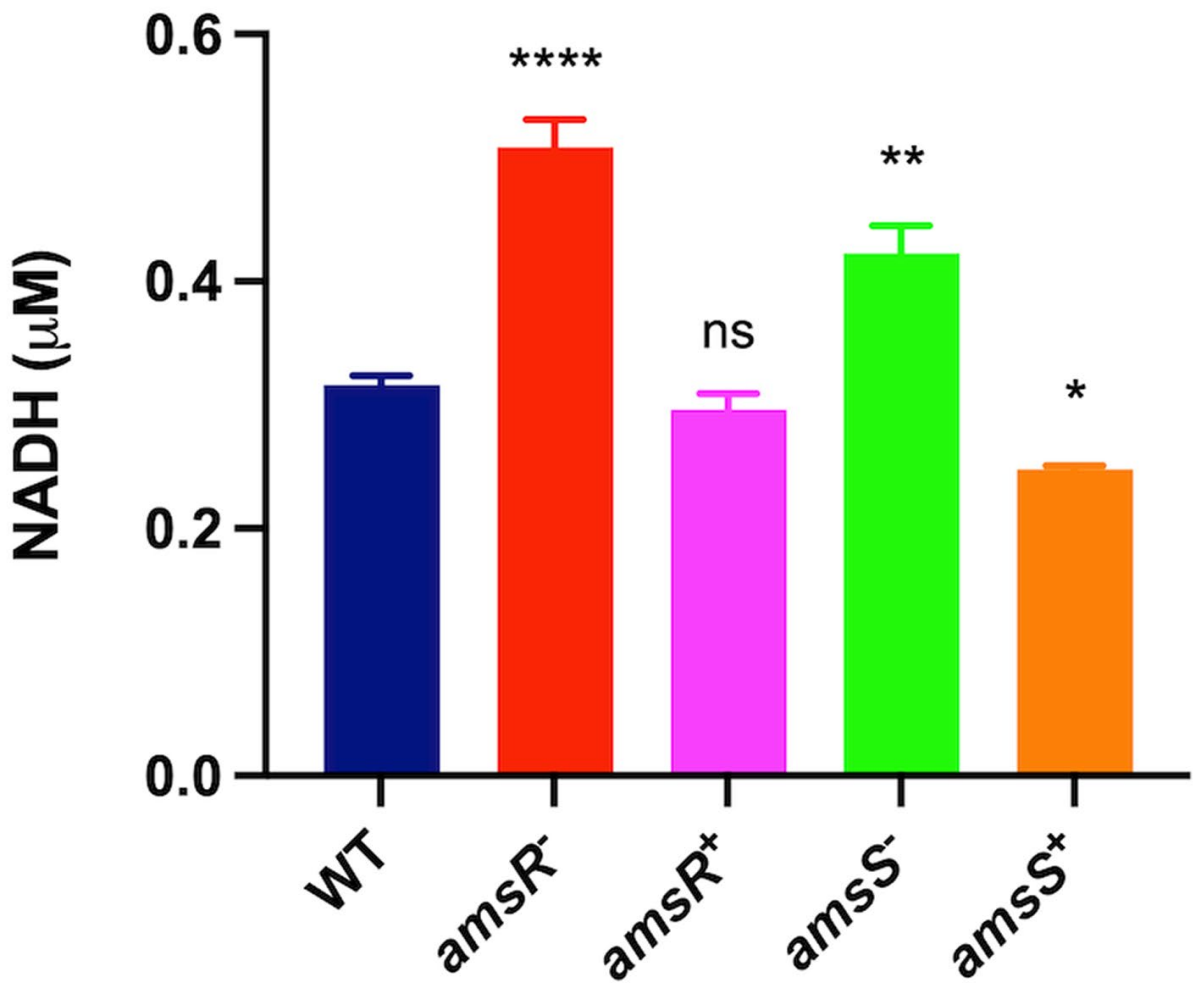
Disruption of AmsSR results in elevated intracellular NADH levels. Intracellular levels of NADH were measured in exponentially growing cells. Data is derived from three independent biological replicates. Error bars ± SEM. One-way analysis of variance, with Dunnett’s multiple-comparisons test, was used to assess statistical significance in comparison to wild-type, ^*^p < 0.05, ^**^p < 0.01, ^****^p < 0.0001, ns, not significant.

Disruption of *amsSR* leads to increased conversion of pyruvate to acetyl-coA. Furthermore, during the glyoxylate shunt, malate is converted to oxaloacetate by the enzyme malate dehydrogenase. These reactions have the side effect of generating NADH as a byproduct (Figure 5). As such, one might hypothesize that our mutants would have increased levels of NADH. Accordingly, we next measured NADH levels in our strains (Figure 9). We determined that the mutants had significantly higher levels of NADH (*amsR*^−^ = 0.51 μM, *amsS*^−^ = 0.42 μM) compared to the wildtype (0.32 μM) and complemented strains (*amsR^+^* = 0.30 μM, *amsS^+^* = 0.25 μM). This implies that the increased NADH in our mutants may be the result of hyperactive TCA enzymes generating NADH. Additionally, the excess NADH produced in mutants cannot be oxidized *via* the ETC, as we observed significant reduction in expression of numerous components of this system, including: the succinate dehydrogenase complex (complex II), which has distinct roles in both the TCA cycle and the ETC; a gene encoding part of the cytochromes b_c1_ complex III, which has high affinity for oxygen; *cyoE* a component of the Complex IV terminal oxidase cytochrome; and genes within the ATP generation machinery: *atpH, atpB*, and *atpE* (Park et al., 1995; Deckers-Hebestreit and Altendorf, 1996). Together this data supports a disruption of the respiratory chain in our mutants that likely results in decreased electron flow in the presence of glucose.

### *amsSR* mutants favor ATP production *via* substrate phosphorylation

**Figure 10:**
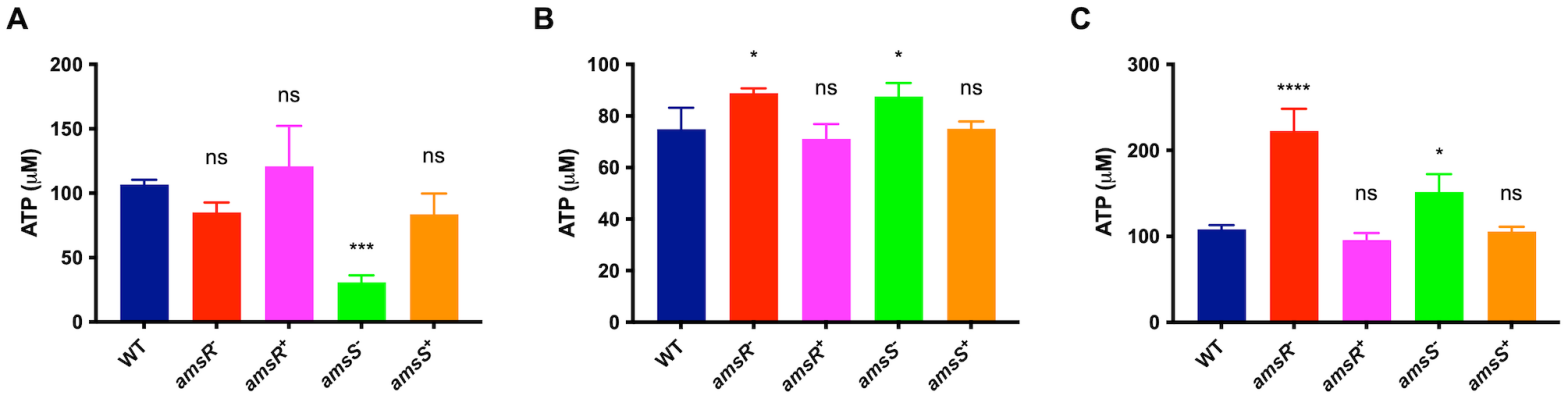
*amsSR* mutants favor ATP production *via* substrate phosphorylation. Intracellular ATP levels were measured in exponentially growing cells. Strains were grown in: **(A)** LB containing pyruvate (10 mM), **(B)** LB containing CCCP (6.25 μg/mL), or **(C)** LB containing both CCCP (6.25 μg/mL) and pyruvate (10 mM). Data is derived from three independent biological replicates. Error bars ± SEM. One-way analysis of variance, with Dunnett’s multiple-comparisons test, was used to assess statistical significance in comparison to wild-type, ^*^p < 0.05, ^***^p < 0.001, ^****^p < 0.0001, ns, not significant.

The generation of ATP occurs *via* two processes: oxidative phosphorylation and substrate level phosphorylation, which is dependent on the partial oxidation of metabolites (Rose et al., 1954; Amarasingham and Davis, 1965; Richardson, 2000). Given that we observed decreased intracellular pH and membrane depolarization, as well as a seemingly impaired ETC, one might hypothesize that oxidative phosphorylation would be diminished upon *amsSR* disruption. To test this, we determined ATP levels in the wild-type, mutants, and complemented strains. First, we quantified ATP levels in the presence of pyruvate only, which, during aerobic growth, is converted to acetyl-CoA before being oxidized *via* the TCA cycle, resulting in the donation of electrons to the ETC; thus, feeding both pathways for ATP generation. Here, we noted that ATP levels were significantly reduced in the *amsS*^−^ mutant (30.7 μM) and slightly reduced for the *amsR*^−^ mutant (85.0 μM), compared to the WT (106.6 μM) and complemented strains (*amsR ^+^* = 121.1 μM, *amsS^+^* = 83.6 μM; Figure 10A), suggesting that our mutants generate ATP *via* substrate level phosphorylation. We next hypothesized that inhibition of the ETC would result in increased ATP levels for our mutants. As such, we repeated these studies in the presence of CCCP. Here (Figure 10B), ATP levels were significantly higher for our mutants (*amsR*^−^ = 88.8 μM, *amsS*^−^ = 87.6 μM) compared to the wild-type (74.9 μM) and complemented strains (*amsR*^+^ = 71.1 μM, *amsS*^+^ = 75.0 μM). Moreover, after treatment with CCCP and pyruvate, we found that the mutants again demonstrated higher levels of ATP (*amsR*^−^ *=* 220.4 μM, *amsS*^−^ *=* 151.7 μM) compared to the wildtype (108.3 μM) and complemented strains (*amsR^+^* = 95.5 μM, *amsS^+^* = 105.1 μM; Figure 10C). As such, when ATP generation *via* oxidative phosphorylation is abolished (by addition of CCCP) our mutants actually generate more ATP than the wild-type and complemented strains. Collectively, this suggests that our mutants have an impaired capacity to produce ATP *via* oxidative phosphorylation but compensate for this by enhanced ATP generation by upregulating fermentative pathways.

### Reduced oxygen consumption in the mutants indicates altered aerobic respiration

**Figure 11:**
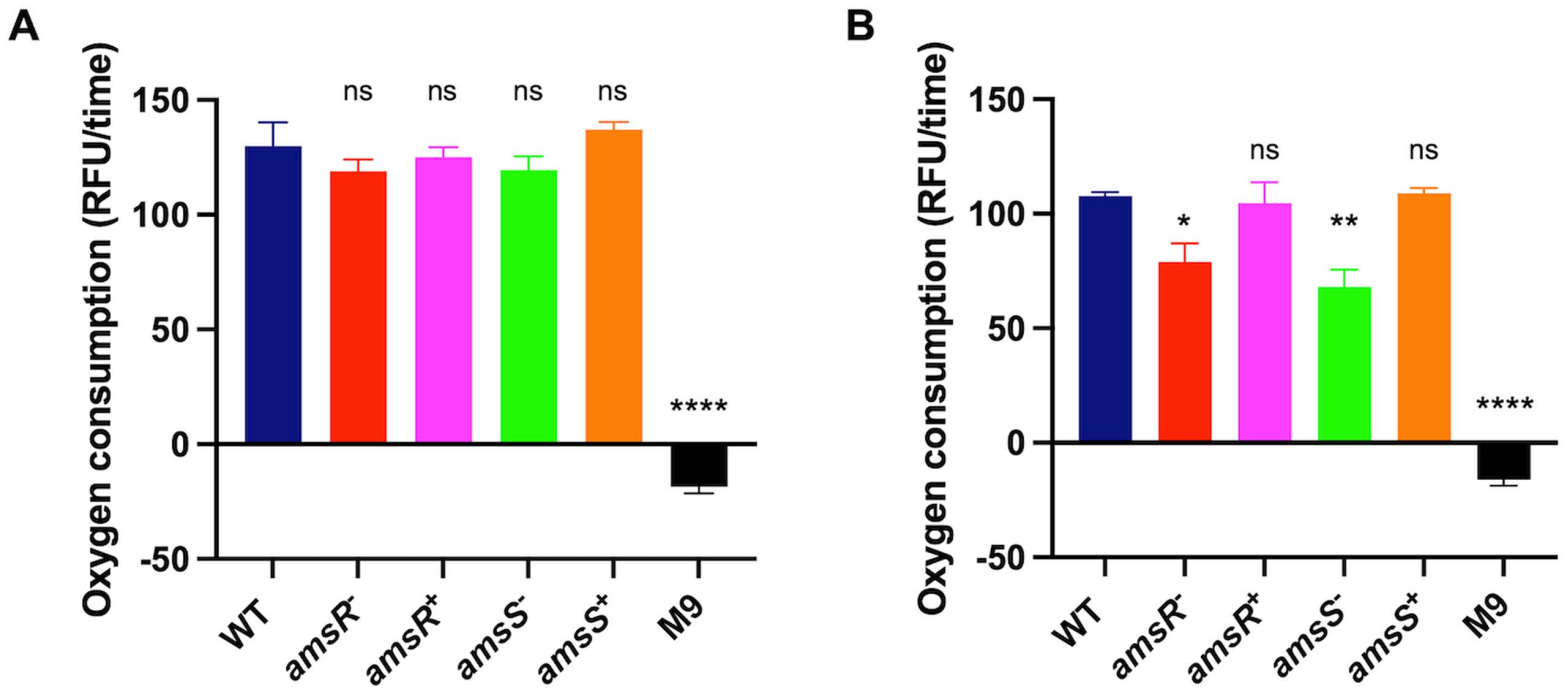
*amsSR* mutants consume less oxygen due to a dysregulated electron transport chain. Extracellular O_2_ consumption was measured in strains diluted to an OD_600_ of 0.1 in: **(A)** M9 media supplemented with 1% casamino acids; or **(B)** M9 media supplemented with 1% casamino acids and 0.2% glucose. All strains were then exposed to 10 μL of the O_2_ consumption reagent, with relative fluorescence measured in black 96-well plates sealed with 100 μL of mineral oil. The rate of oxygen consumption was calculated after 30 min. Data is derived from three independent biological replicates. Error bars ± SEM. One-way analysis of variance, with Dunnett’s multiple-comparisons test, was used to assess statistical significance in comparison to wild-type, ^*^p < 0.05, ^**^p < 0.005, ^****^p < 0.0001.

Our findings suggest that *amsSR* mutants have increased production of ATP *via* substrate phosphorylation, while generating lower ATP levels *via* oxidative phosphorylation. This effect is likely mediated *via* an impaired ETC, which would hinder ATP production. This is in agreement with our RNA seq, which shows decreased expression of genes encoding components of the ETC in our mutant, including succinate dehydrogenase (complex II); cytochrome *b*_561_, (*cybB*, ABUW_2753), which encodes a respiratory b-type oxidase involved in the transfer of electrons to quinones; a CioAB type oxidase (ABUW_2389, *cioA*; ABUW_2390, *cioB*); and the ATP generation machinery (Lundgren et al., 2018). This is also accompanied by a lack of change in intracellular pH in the presence of glucose for the mutants. During aerobic respiration, glucose is oxidized *via* the TCA cycle, generating electrons that are transferred to the ETC. The flow of electrons through the ETC is coupled to translocation of H^+^, which is then utilized by the ATP machinery, ultimately resulting in increased oxygen consumption (Henkel et al., 2014). Given that our *amsSR* mutants appear to have altered activity of the ETC, one would expect altered oxygen consumption rates in our mutant strains. To test this, we monitored oxygen consumption in our strains in the presence or absence of glucose. We determined that, in the absence of glucose, no differences were observed between our various strains (Figure 11A). In contrast, in the presence of glucose the mutant strains displayed decreased oxygen consumption compared to the wild-type and complemented strains (Figure 11B). As such, these results suggest that disruption of *amsSR* affects the function of the aerobic respiratory chain, impacting the energy status of the cell, which in turn upregulates alternative metabolic and energy generation pathways in our mutants.

## Discussion

In this study we follow up our previous work exploring regulatory networks in *A. baumannii*, identifying the novel TCS, AmsSR. Given that AmsS is so unusual in structure, containing 14 TMD, we elected to explore how conserved this TCS was among other Acinetobacter species. At present, there are 100 proposed species of Acinetobacter on the LPSN (Supplementary Table S3; Parte et al., 2020). We performed BLAST analysis at EnsemblBacteria for 55 of these for which genome sequences were present (Supplementary Table S3). Interestingly, only 17 organisms returned high confidence homologs to AmsS with the expected 14 TMDs and hybrid HK structure; while the remaining 38 did not (all hits in these other organisms were low homology hits to GacS type HKs, which only has 2 TMDs). Strikingly, AmsS proteins share significant amino acid sequence identity (Supplementary Table S4) and relatedness (Supplementary Figure S4) throughout the entire protein (Supplementary Figure S5). Of note, the *Acinetobacter calcoaceticus*–*Acinetobacter baumannii* complex (Villalon et al., 2019) had very high sequence identity (dark green, Supplementary Table S4), which is perhaps to be expected. Two other organisms, *A. puyangensis* and *A. populi* appear to form another group of high sequence identity; as did *A. schindleri*, *A. lwoffi* and *A. idrijaensis*, although there is only a single amino acid difference in the AmsS of these latter two organisms, suggesting a potential misidentification of species for these genomes. Notably, the AmsS from *A. rudis* had the lowest homology to other species (red), but still retained > 55% identity. Outside of *Acinetobacter* species, we also found AmsS homologs in a few other organisms, including *Alkanindiges hydrocarboniclasticus*, *Paraburkholderia sprentiae* and *Pseudomonas kuykendallii*. Although these proteins have lower homology to AmsS from *A. baumannii* (Supplementary Table S6), they all still retain the 14 TMD arrangement, alongside the hybrid HK structure (not shown). The seeming sporadic conservation of AmsSR in *Acinetobacter* species, and more widely across the betaproteobacteria and gammaproteobacteria is both puzzling and fascinating. The exact reason for this is not clear, but deeper exploration of the metabolic capacities and pathways of AmsSR containing organisms may shed further light.

When considering locus organization of *amsSR* in *A. baumannii* (Supplementary Figure S1), we note that the two genes overlap by 4 nucleotides. Although such an arrangement is not uncommon, it typically suggests that the genes in question are transcriptionally linked. Thus, in these studies we have presumed a single promoter, upstream of *amsS*, drives expression of both genes. It is noteworthy then that phenotypes associated with *amsS* inactivation can be complemented by *amsS* alone, and do not require *amsR*. To explore this, we performed RT-qPCR for *amsR* in the wild-type strain and *amsS* mutant. Here we demonstrate that *amsR* is transcribed within the *amsS*^−^ mutant in both the presence and absence of CCCP (Supplementary Figure S6) at levels greater than the wild-type. Furthermore, we performed sequence analysis at the 3′ end of *amsS* and identified a σ^A^ like promoter close to the *amsR* translation initiation regions (Supplementary Figure S7). Thus, either there are promoter elements on the transposon that facilitate read through for *amsR* expression in the *amsS* mutant, or there is a feedback loop whereby the loss of AmsS activity upregulates the putative *amsR* promoter. Further investigation is required to determine which of these scenarios is true, however these findings explain why we are able to complement the *amsS* mutant with *amsS* alone.

Recently, a publication appeared that partially characterized *amsSR* in the clinical isolate 04117201 (Giles et al., 2022). In that study, it was shown that *AmsSR* (named StkRS therein; an abbreviation of sticky) influenced colistin resistance through regulation of *pmrCAB*. However, the *stkR* mutants used contained multiple unintended mutations in their genome as compared to the wild-type. Additionally, no complementation was presented in this study. Given that the Stk abbreviation is already widely used to refer to Serine/Threonine Kinases, and that our data indicates a robust function for this TCS, we instead use our own more descriptive name for AmsSR (Regulator of Alternative Metabolic Systems).

A key finding of our study was upregulation of P*_amsSR_* in the presence of CCCP. CCCP bypasses the ATP synthase-dependent active transport of protons into the cytoplasm by increasing membrane permeability to H^+^. As a result, activity of the ATP synthase and ATP synthesis in general halts, leading to disruption of the ETC. This has the effect of limiting the transfer of electrons to quinones, altering the energy status of the cell (Burstein et al., 1979; Ghoul et al., 1989; Bekker et al., 2007). To explore this, we tested the impact of other uncouplers, with distinct mechanisms of action toward membrane energetics, on P*_amsSR_* activity. These included DCCD, valinomycin, and DNP; none of which altered the expression of this TCS. Interestingly, in a study on the *S. aureus lrgAB* operon, which regulates cell death, was shown to be induced only by CCCP and gramicidin despite testing other proton uncouplers (nigericin and DCCD; Patton et al., 2006). When assessing the effects each proton uncoupler had on membrane potential, they found that only CCCP and gramicidin completely depolarized membranes, while both nigericin and DCCD depolarized the membrane by around half or had little effect on the membrane potential, respectively. Interestingly, valinomycin alone resulted in hyperpolarization and did not induce the expression of *lrgAB*. While the proton uncoupler DNP was not tested in this study, the inability of this compound to induce P*_amsSR_* in *A. baumannii* would also suggest that it too does not depolarize the membrane. These findings are perhaps unsurprising when one considers the specific effects each uncoupler has on the cell. For example, DCCD inhibits ATPase activity without reducing electron flow through the ETC (Clejan et al., 1984; Degli Esposti and Lenaz, 1985; Shinkarev et al., 2000). Similarly, the disruption of membrane potential caused by valinomycin has little impact on oxygen consumption (Azarkina and Konstantinov, 2002). Finally, DNP accelerates respiration by stimulating complete oxidation of intermediate metabolites such as succinate and fumarate (Clifton and Logan, 1939; Clifton, 1966). Given that none of these agents induce activity of P*_amsSR_*, the specific response of this TCS to CCCP could be explained by its targeted effects on quinones pools, as it is the only uncoupler that inhibits hydrogen pumping by cytochrome c, disrupting the electrochemical gradient (Bogachev et al., 1995; Otten et al., 1999).

The effects of CCCP impacting energy generation are similar to those observed during the transition from aerobic to anaerobic growth, a process governed in *E. coli* by the TCS ArcAB (Iuchi and Lin, 1988; Iuchi et al., 1989). As oxygen becomes limited, a reduction in energy generation *via* the respiratory chain results in reduced quinone pools. This is sensed by two conserved cysteine residues within the HK, ArcB, which are oxidized when quinone pools are plentiful. Consequently, quinone depletion *via* diminished oxygen levels leads to reduction of these cysteine residues, activating the HK. This leads to activation of the ArcA RR, which in turn modulates expression of genes involved in alternative energy generations pathways. Despite *A. baumannii* lacking an ArcAB homolog (Casella et al., 2017) during our bioinformatic study of AmsS, we observed that one of the two ArcB cysteines aligns with a conserved cysteine in AmsS (Supplementary Figure S8; Malpica et al., 2004). When looking at other ArcB homologs, we noted that the dual cysteine arrangement was only conserved between *E. coli* and the closely related *S. enterica*, while *V. cholera* retains only one cysteine, and *H. influenzae* and *S. oneidensis* lack both. As such, it is tempting to speculate that AmsS of *A. baumannii* may also senses ubiquinone pools and be regulated by oxidation at its lone cysteine residue, in a manner akin that for ArcB in *E. coli*. Indeed, when exploring the ability of our strains to adapt to oxygen limited conditions, we observed that our mutants displayed a growth defect compared to the wild-type and complemented strains (Supplementary Figure S9).

When we examined the effects of disruption of *amsSR* on membrane energetics, we observed that our mutants exhibited a depolarized membrane in conjugation with decreased intracellular pH levels; indicating a lack of protons being pumped out of the cell that could be used for ATP production. This would suggest that the *amsSR* mutants would be unable to generate energy from glucose. However, our mutants were able to grow in the presence of glucose as a lone carbon source. Of note, *A. baumannii* is unable to assimilate glucose, but must convert it to gluconate *via* PQQ dependent-glucose dehydrogenase proteins. Studies on the regulation of PQQ biosynthesis in the soil bacteria *P. putida* and *M. extorquens* found that expression of *pqqB* or *ppqF* drive synthesis of PQQ, and that the amount of *pqqA* expression has little effect on the amount of PQQ produced (Toyama and Lidstrom, 1998; An and Moe, 2016). This suggests that despite decreased expression of *pqqA* in the *amsR* mutant, the PQQ levels produced may be sufficient for conversion of glucose to gluconate, as we observe accumulation of pyruvate, which is a product of active glycolysis.

Pyruvate is further oxidized *via* the TCA cycle, which generates NADH. NADH is then oxidized by the ETC allowing for production of ATP *via* oxidative phosphorylation. The absence of change in intracellular pH in the presence of glucose in our mutants suggests that the loss of AmsSR reduces activity of the respiratory chain, which translates into diminished electron flow and active pumping of H^+^. In line with this, we observed that our mutants have decreased expression of genes encoding components of the respiratory chain irrespective of glucose oxidation, as well as increased intracellular NADH levels. These observations are similar to that previously documented for other ubiquinone sensing TCS mutants (Alexeeva et al., 2000; Park et al., 2013). Furthermore, the overproduction of NADH in *E. coli* is thought to exert allosteric control on the activity of succinate dehydrogenase (complex II), likely interrupting electron flow from the TCA cycle to the respiratory chain (Levanon et al., 2005). Each of these changes leads to alterations that mirrors those caused by CCCP, disrupting the respiratory chain, and altering the redox state of quinone pools. Thus, loss of AmsSR function in our mutant strains would render them unable to sense such changes and leave them incapable of responding by regulating genes that are required for the restoration of membrane energy.

Further to this, the disruption in succinate dehydrogenase activity, due to elevated levels of NADH, results in replacement of the TCA cycle with the glyoxylate shunt. For heterotrophic bacteria, the glyoxylate shunt is vital for their survival in iron limiting conditions (Debeljak et al., 2019). This is because iron is an essential co-factor for enzymes inside the ETC, thus the lack of iron hinders the ability of heterotrophic bacteria to generate ATP. Therefore, the activation of the glyoxylate shunt helps circumvent the loss of the ETC similar to that which we observe for our mutants. Moreover, a study investigating transcriptional changes of *E. coli* switching from anaerobic to aerobic conditions found upregulation of TCA cycle genes, terminal oxidases with high affinity to oxygen, and an abundance of reduced equivalents for the respiratory chain. Thus, the altered expression of genes involved in glycolysis and the TCA cycle in our *amsR* mutant further support a rewired energy generation network in this strain.

The *pta-ackA* operon was also significantly upregulated in our *amsR* mutant. This pathway is activated in response to increased levels of glucose, oxygen, and high metabolic activity, and the conversion of pyruvate to acetyl-CoA under aerobic conditions (Wolfe, 2005; Vemuri et al., 2006). Conversely, this pathway produces mixed-acid products, lactate, ethanol, and acetate under anaerobic conditions (Enjalbert et al., 2017). A hallmark of *Acinetobacter* species is their ability to grow on acetate as a sole carbon source; thus, it is possible that our mutants respond to CCCP-dependent changes in the redox state of quinone pools with a hyperactive glyoxylate shunt that causes carbon overflow, diverting to the Pta-AckA pathway. Notably, we observed higher levels of pyruvate and acetate in our mutants that could likely be explained by the increased expression of genes encoding pyruvate dehydrogenases and the Pta-AckA pathway (Akhova and Tkachenko, 2014; Enjalbert et al., 2017). Collectively, our data demonstrates that the increase in metabolic overflow in our mutants result in saturation of the main energy generation pathways of glycolysis and the TCA cycle. Therefore, this would suggest that ATP generation *via* substrate level phosphorylation prevails to compensate for a reduced ability to generate ATP *via* oxidative phosphorylation. Interestingly, it has been reported that *E. coli* switches to using fermentation pathways over metabolic pathways that require oxygen during higher growth rates and overflow metabolism (Szenk et al., 2017). This is thought to be in part due to the lower biosynthetic cost associated with fermentation in comparison to using the ETC (Basan et al., 2015) Therefore, the increased growth rates displayed by the *amsS*^−^ and *amsR*^−^ mutants when glucose is used as a sole carbon source would be further indication that they rely on substrate level phosphorylation for energy production, since this would lower the biosynthetic costs for producing ATP.

In summary, our data demonstrates the importance of AmsSR in controlling expression of *A. baumannii* metabolic pathways. We reveal that loss of AmsSR in this obligate aerobe leads to altered respiratory chain function, and consequential metabolic overflow. This ultimately results in altered metabolic pathway utilization, affecting energy generation—highlighting the importance of AmsSR in maintaining essential energy homeostasis.

## Data availability statement

The datasets presented in this study can be found in online repositories. The names of the repository/repositories and accession number(s) can be found in the article/Supplementary material.

## Author contributions

LS and LC conceptualized the study. LC, NT, and BT performed the experiments. LS, LC, NT, BT, and MS analyzed the data. LC wrote the original draft. LS, NT, and MS edited the manuscript. All authors contributed to the article and approved the submitted version.

## Funding

This work was supported by grant AI124458 (LS) from NIAID. This work has also been supported by the USF Genomics Core.

## Conflict of interest

The authors declare that the research was conducted in the absence of any commercial or financial relationships that could be construed as a potential conflict of interest.

## Publisher’s note

All claims expressed in this article are solely those of the authors and do not necessarily represent those of their affiliated organizations, or those of the publisher, the editors and the reviewers. Any product that may be evaluated in this article, or claim that may be made by its manufacturer, is not guaranteed or endorsed by the publisher.
