# Supplementary material for: The novel two-component system AmsSR governs alternative metabolic pathway usage in Acinetobacter baumannii
Source: Front Microbiol. 2023 Apr 4;14:1139253. doi: 10.3389/fmicb.2023.1139253 (PMC10112286; doi:10.3389/fmicb.2023.1139253)
Supplement: Supplementary file 1 [file Data_Sheet_1.PDF]

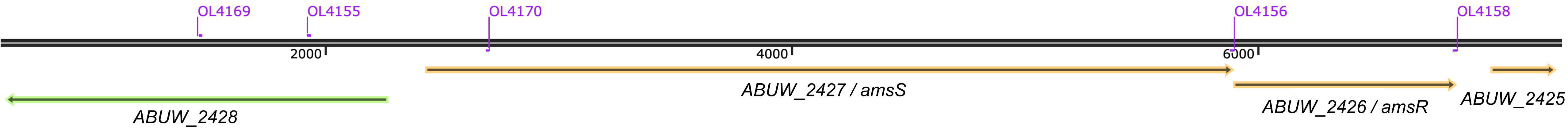

Figure S1. Schematic of the AmsSR Locus. Shown is the *asmSR* (ABUW_2427-2426) coding region, drawn to scale, with surrounding sequences. Denoted in purples are primers used in this study. Image created using Snapgene.

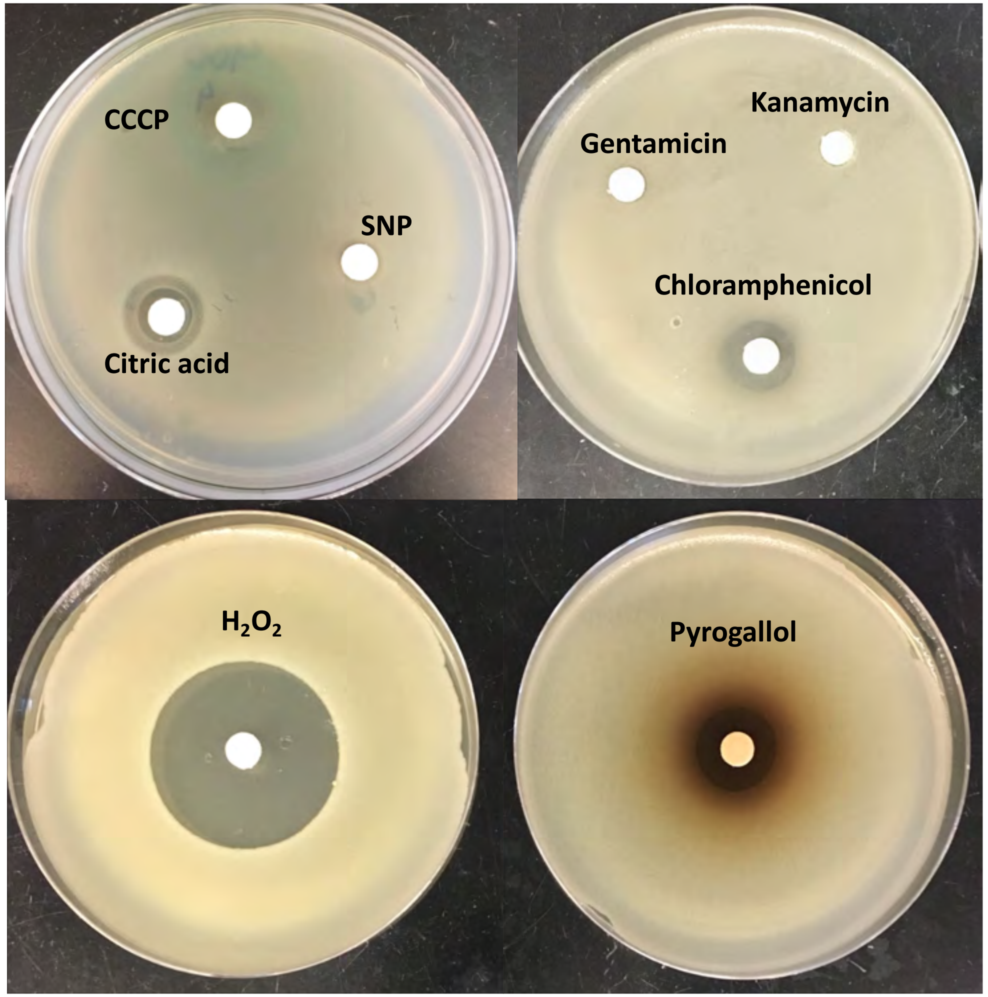

Figure S2. The *amsSR* promoter is induced upon exposure to CCCP. LB plates supplemented with X-Gal were inoculated with the wild-type bearing an *amsSR-lacZ* reporter-gene fusion. Sterile filter disks were placed onto plates and inoculated with the compounds noted. The induction of expression is visualized as a blue ring around the zone of inhibition. Shown are four representative plates.

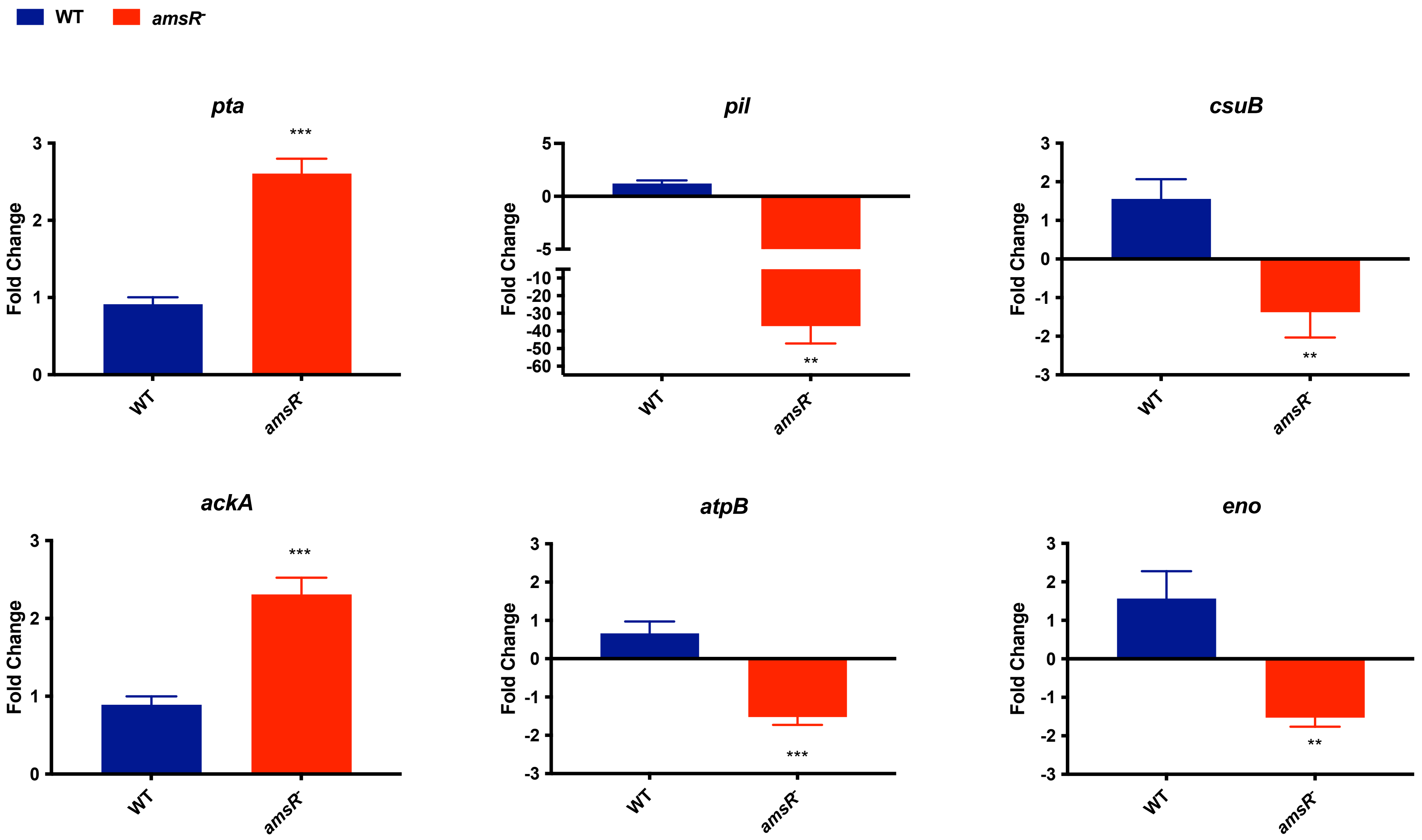

Figure S3. qPCR validation of RNA-seq data. Transcriptional changes from the RNA-seq dataset were confirmed in the *amsR* mutant and wild-type strain using qRT-PCR analysis for a random selection of genes. Measurements are derived from three independent replicates with error bars shown ±SEM. Student’s t-test was used to assess statistical significance ** = p<0.001, *** = p<0.001.

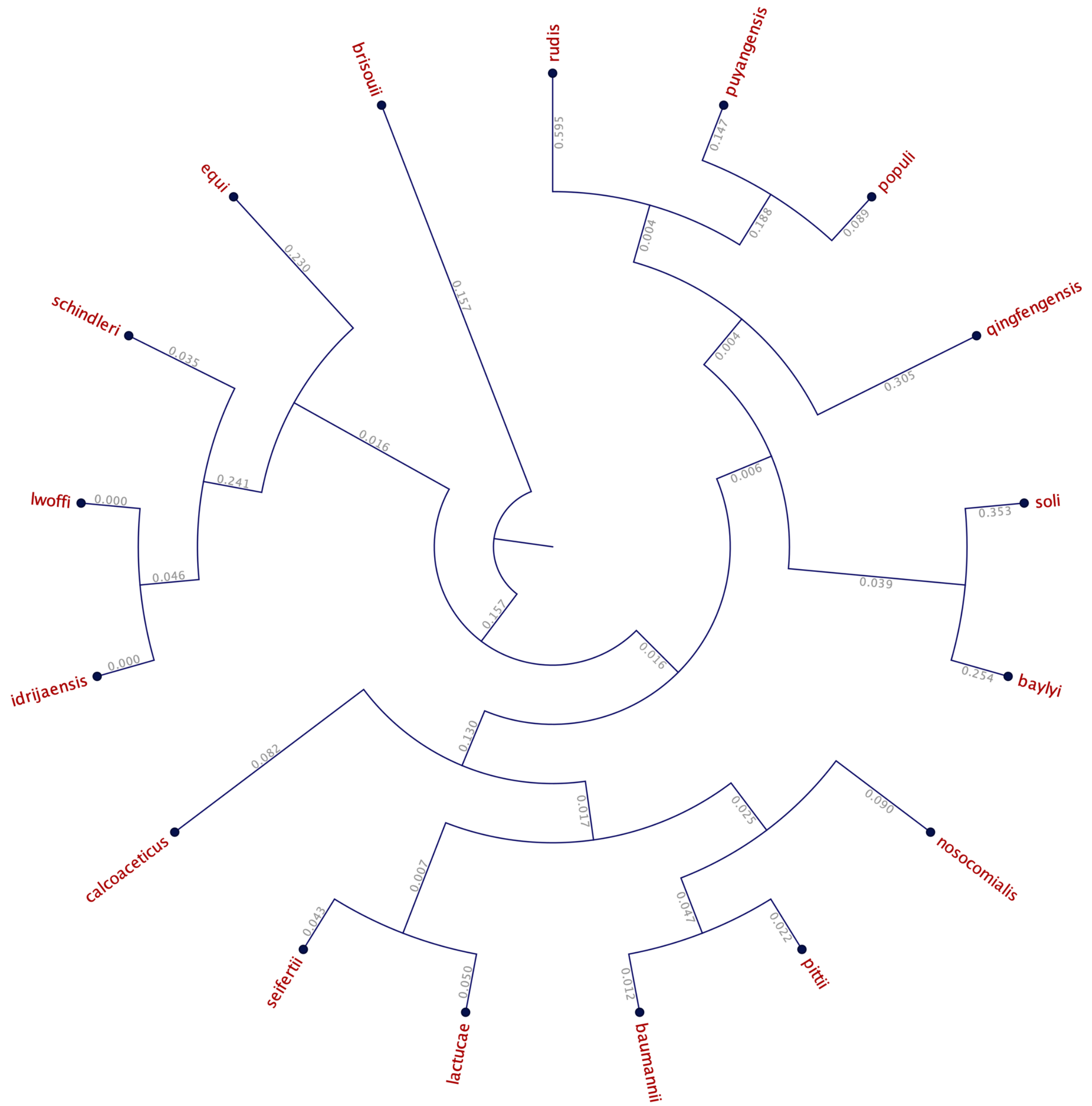

Supplemental Figure S4. The AmsS homologs of Acinetobacter species display a high degree of relatedness. Shown is a circular cladogram of Acinetobacter AmsS homologs from Table S4. Image generated using Qiagen CLC Main Workbench (CLC bio, v 21.0.1). Numbers refer to branch lengths.

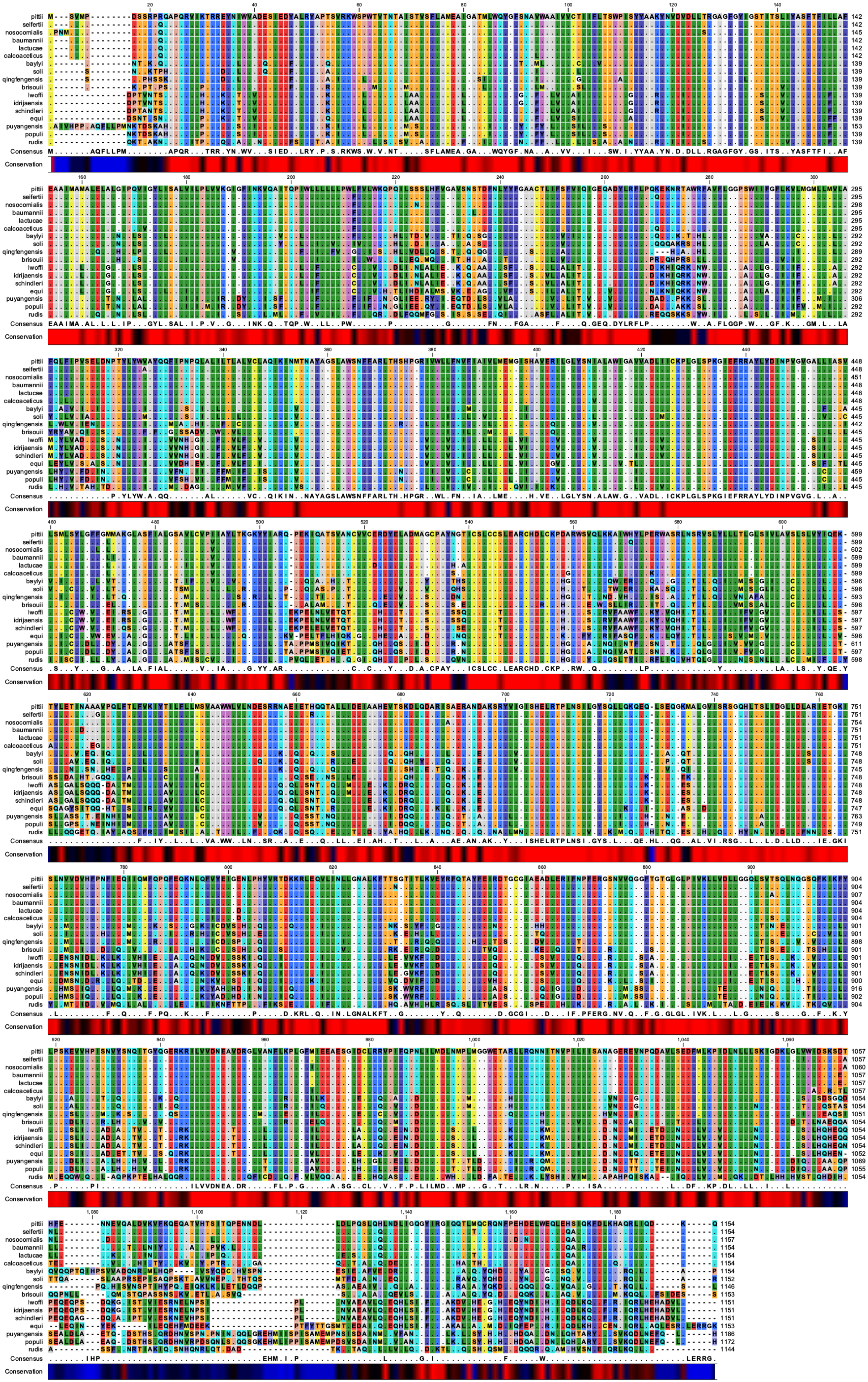
pittii
seifertii
nosocomialis
baumannii
lactucae
calcoaceticus
baylyi
soli
qingfengensis
brisouii
lwoffi
idrijaensis
schindleri
equi
puyangensis
populi
rudis
Consensus
Conservation

Supplemental Figure S5. Protein alignments of AmsS Homologs from *Acinetobacter* species. Alignments of AmsS proteins from Table S4 / Figure S4 generated using Qiagen CLC Main Workbench (CLC bio, v 21.0.1). Conservation colors range from red (100%) to blue (0%). Identical residues are shown as the same color and a "." rather than duplicating the same amino acid letter.

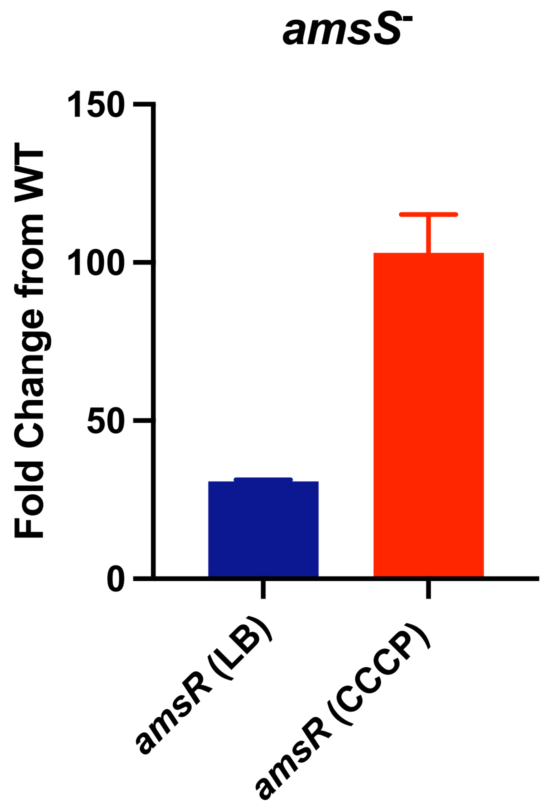

Supplemental Figure S6. *amsR* is transcribed within the *amsS*⁻ mutant. *amsR* transcription within the *amsS*⁻ mutant was confirmed using qRT-PCR analysis. The wild-type strain and *amsS*⁻ mutant were grown, and RNA was extracted, as described for the RNA-seq experiments. Measurements are derived from three biological replicates with error bars shown ±SEM.

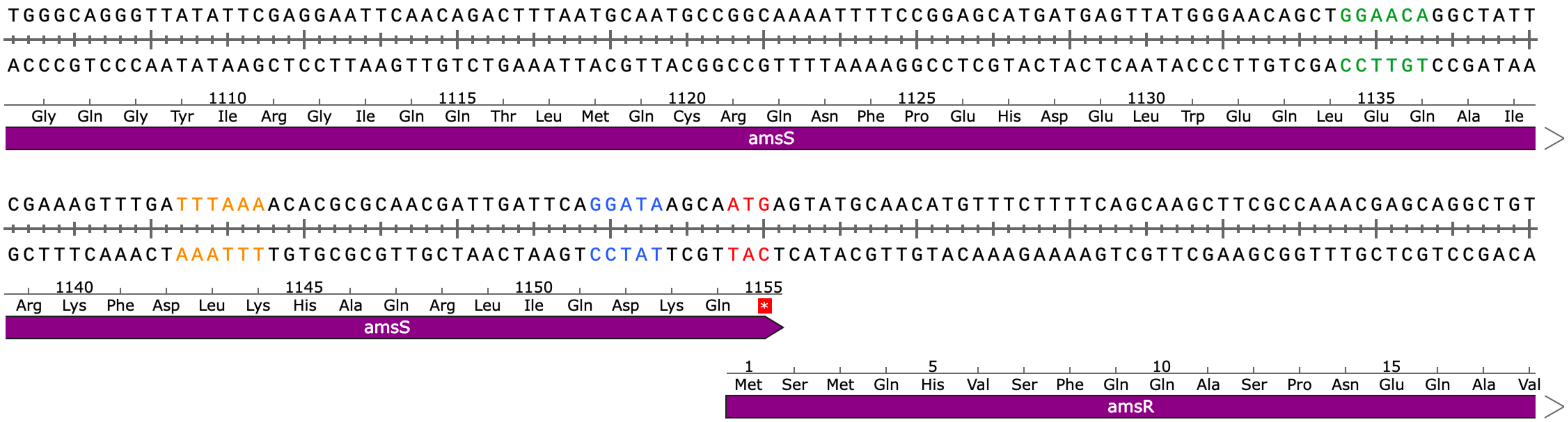

Supplemental Figure S7. Putative *amsR* promoter. A putative promoter for *amsR* was identified within the *amsS* ORF. The red colored nucleotides denote the start codon for *amsR*, blue colored nucleotides denote the ribosomal binding site, and the orange and green colored nucleotides indicate the -10 and -35 sites of the promoter, respectively. Image created using Snapgene.

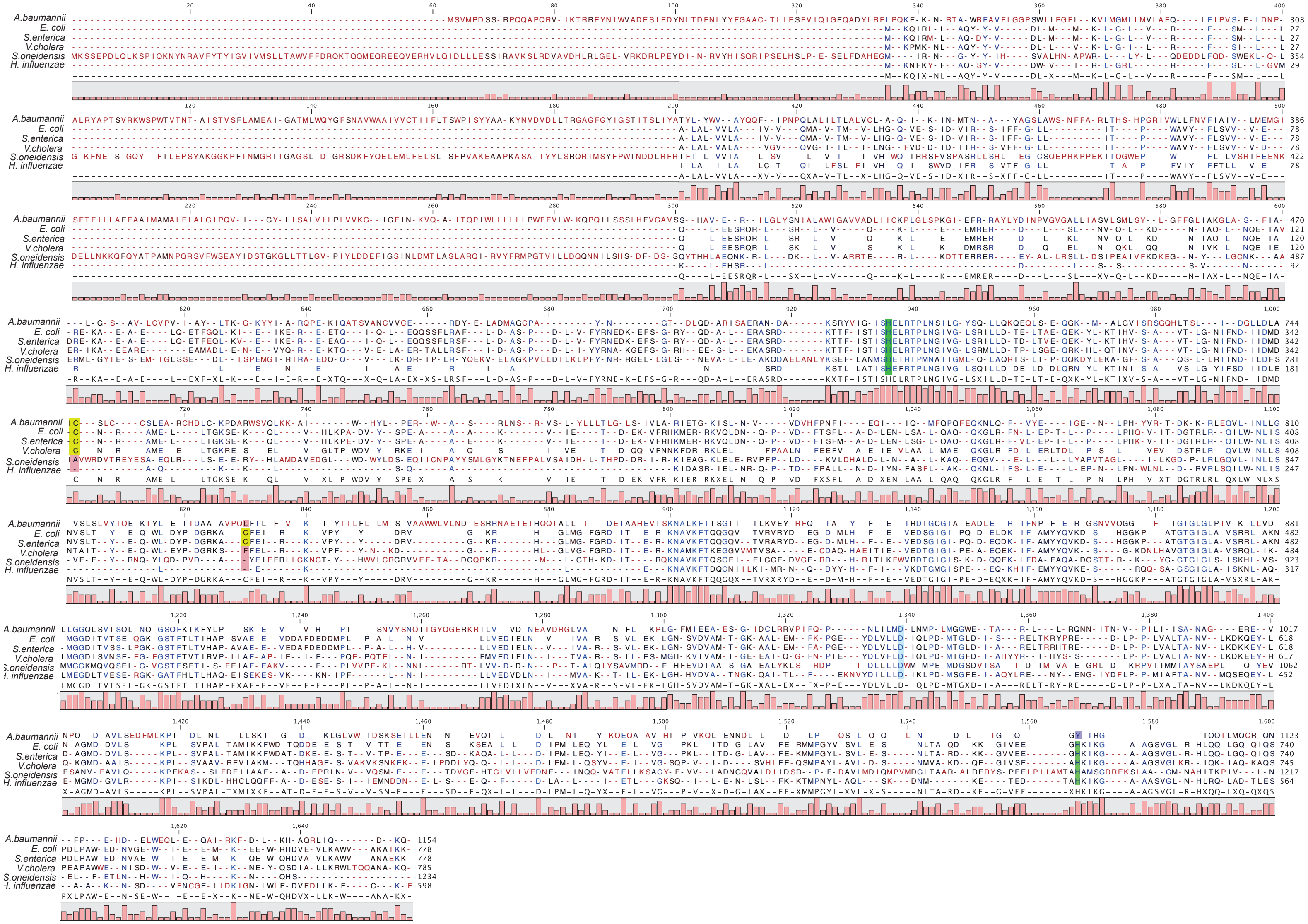

Supplemental Figure S8. Protein alignments of AmsS from *A. baumannii* alongside ArcB proteins from a variety of organism. Alignments of AmsS of *Acinetobacter baumannii* and ArcB protein sequences from *Escherichia coli*, *Salmonella enterica*, *Vibrio cholera*, *Shewanella onidensis*, and *Haemophilus influenzae* showing conservation and divergence of amino acids that are required for ArcB kinase regulation in *E. coli*, whilst pink shading shows divergence of these residues. Blue shading shows conservation of the aspartic acid residue. Green shading shows conservation of histidine residues, with violet indicating the absence of the second of these in *A. baumannii*.

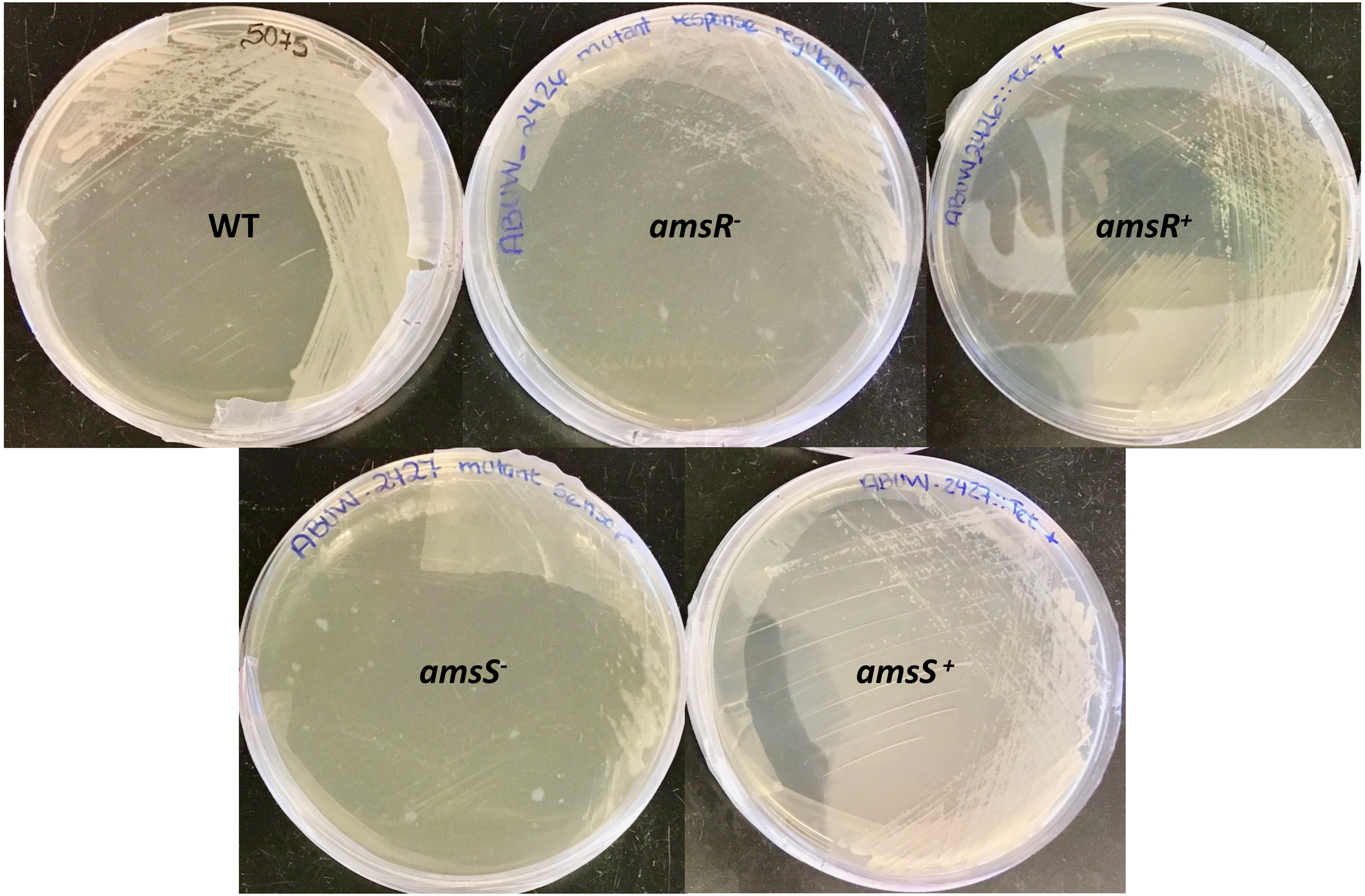

Figure S9. *A. baumannii amsSR* mutants display growth defects under oxygen limiting conditions. The wild-type, *amsS* and *asmR* mutants, alongside their complementing strains were grown on LB agar incubated at 37 °C for 1 week in a GasPak EZ Incubation Container with GasPak EZ Anaerobe Sachets to restrict oxygen availability.
